# A randomized controlled trial of Golden Ratio, Feng Shui, and evidence based design in healthcare

**DOI:** 10.1371/journal.pone.0303032

**Published:** 2024-06-05

**Authors:** Emma Zijlstra, Bart van der Zwaag, Sabine Kullak, Ab Rogers, Dean Walker, Sjoukje van Dellen, Mark Mobach

**Affiliations:** 1 Research Group Facility Management, Hanze University of Applied Sciences, Groningen, The Netherlands; 2 International Feng Shui Association, Singapore, Singapore; 3 Ab Rogers Design, London, United Kingdom; 4 Department of Psychology, University of Groningen, Groningen, The Netherlands; 5 Research Group Spatial Environment and the User, The Hague University of Applied Sciences, The Hague, The Netherlands; Public Library of Science, UNITED KINGDOM

## Abstract

**Background:**

In a global effort to design better hospital buildings for people and organizations, some design principles are still surrounded by great mystery. The aim of this online study was to compare anxiety in an existing single-bed inpatient hospital room with three redesigns of this room in accordance with the principles of Golden Ratio, Feng Shui, and Evidence-Based Design.

**Methods:**

In this online multi-arm parallel-group randomized trial participants were randomly assigned (1:1:1:1) to one of four conditions, namely Golden Ratio condition, Feng Shui condition, Evidence-Based Design condition, or the control condition. The primary outcomes were anxiety, sense of control, social support, positive distraction, and pleasantness of the room.

**Findings:**

Between June 24, 2022, and August 22, 2022, 558 individuals were randomly assigned to one of the four conditions, 137 participants to the control condition, 138 participants to the Golden Ratio condition, 140 participants to the Feng Shui condition, and 143 participants to the Evidence-Based Design condition. Compared with baseline, participants assigned to the Evidence-Based Design condition experienced less anxiety (mean difference -1.35, 95% CI -2.15 to -0.55, Cohen’s d = 0.40, p < 0.001). Results also showed a significant indirect effect of the Feng Shui condition on anxiety through the pleasantness of the room (B = -0.85, CI = -1.29 to -0.45) and social support (B = -0.33, CI = -0.56 to -0.13). Pleasantness of the room and social support were mediators of change in anxiety in the Evidence-Based Design and Feng Shui conditions. In contrast, application of the design principle Golden Ratio showed no effect on anxiety and remains a myth.

**Interpretation:**

To our knowledge, this is the first randomized controlled trial linking design principles directly to anxiety in hospital rooms. The findings of our study suggest that Feng Shui and Evidence-Based Design hospital rooms can mitigate anxiety by creating a pleasant looking hospital room that fosters access to social support.

**Clinical trial registration:**

The trial is registered with ISRCTN, ISRCTN10480033.

## Introduction

In a long tradition of design principles, some are still surrounded by great mystery. Well-known examples are the Golden Ratio and Feng Shui. In a global effort to design better hospital buildings for people and organizations, designers may benefit from a demystification of these principles. Given the high stress of illness and recovery, patients are especially likely to benefit from appropriate designs. It is expected that practice application can be improved by clarification of its impact, for instance, on human experience and health.

This study compares the impact of three design principles on patients: two classical and one contemporary. The three respective design principles are based on mathematics (Golden Ratio), energy (Feng Shui), and statistics (Evidence-Based Design). Much has been written about these principles, but what exactly are the actual effects of spaces that are designed in accordance with these principles? Can such spaces improve health? To assess the effects on people and differences between these design principles, we developed a digital twin of a real-life single-bed inpatient hospital room (see images Fig 2) and adapted this room in accordance with these three design principles.

The Golden Ratio (GR) is a number defined by Euclid of Alexandria in Greece more than 2,000 years ago [1]. The GR is also referred to as the phi number (ϕ), the golden section, or the golden mean. The GR is the proportion between two quantities *a* and *b* when *a*/*b* = *b*/(*a* + *b*), than *a* must be approximately 61.8% of *b* [2]. The GR can also be converted to other geometrical shapes, including the golden rectangle, golden angle, or the golden spiral. A golden rectangle is a rectangle of which the length-to-width ratio equals that of the GR. Much has been written about the mathematical ratio (the never ending number 1.6180339887 …) and it seems that it can be found almost anywhere, from the natural world (e.g., plants, animals) to the artificial world (e.g., art, architecture). The GR principle suggests that a design that is designed in accordance with these proportions has pleasant harmonious qualities and can improve aesthetics [1]. Experimental studies on the aesthetics of the GR started in the 19^th^ century and some studies found preferences for the golden rectangle [3–5]. Although scientific evidence has shown that people may prefer the golden rectangle, the evidence is fragile and often not unambiguous [6]. To date and to our knowledge, no scientific evidence clarifies the influence of the GR on people’s health.

Feng Shui (FS) is incorporated in many aspects of life in Chinese culture. FS is a principle that suggests that a natural energy, the Qi, which cannot be seen, is a source of health, human harmony, and prosperity [7]. The theory of the five elements is the basis of all Chinese metaphysics and states that the elements (i.e., wood, fire, earth, metal, and water) represent all physical (e.g., shapes and materials) and non-physical aspects (e.g., compass directions, seasons) [8]. The flow of Qi is affected by the shapes and forms in the environment, its compass direction, and time. All visible aspects of the environment are part of the FS Form School and this school of thought includes the following four aspects: position, moderation of Qi flow, types of Sha Qi, and arrangement of objects [8]. This can be briefly illustrated with the design of a hospital bedroom. Firstly, the term ‘position’ can be best described in an analogy, as a room that is designed like an armchair. It has a comfortable back (symbol: tortoise), shelters on the left and right (dragon and tiger respectively), and a structure in front (phoenix). These symbols of celestial animals represent the supporting structures of the natural or built environment. FS contends that it is important to establish shelter on both sides of the bed. This is achieved with higher objects on the left side of the bed (dragon) than on the right side of the bed (tiger). Secondly, the ‘moderation of Qi flow’ advises designers to moderate the Qi flow in the room. This flow may bounce within the room, and, for instance, should not rush from door through window or target the patient. In contrast, it needs to accumulate and flow gently to be beneficial for humans. Thirdly, the ‘types of Sha Qi’ refer to properties which impose a negative energy on the patient and should be avoided. Examples of sources of Sha Qi are sharp corners, mirrors, overhead structures, or outside view to traffic and unpleasant objects. Finally, the ‘arrangement’ refers to the position of the bed in relation to doors and functional areas. FS advises to separate calm zones in the room (i.e., bed) from communicative areas (i.e., visitors table) and to position the bed not facing the entrance directly, while still in a position allowing to control the entrance. Again, to date and to our knowledge, no scientific evidence clarifies the influence of FS on people’s health.

Evidence-Based Design (EBD) refers to a design that is guided by empirical evidence of scientifically studied effects of the physical surroundings on people [9]. The evidence is mostly based on research in health care settings. In 1984, *Science* reported that the view on nature from a hospital bed reduced the length of stay, the analgesic intake, and nurses’ evaluative comments of patients [10]. Nowadays, a considerable amount of research is available on evidence-based designs that can improve patient outcomes in hospital environments, and, by doing so, change these into healing environments [11–13]. For example, designers are advised to apply single rooms to reduce hospital acquired infections [14], larger windows with views to calm patients [15], more daylight to reduce stress, pain, and medication use [16], dynamic light to improve sleep [17], less blue-depleted lighting to decrease anxiety [18], or so-called ‘healing art’ to improve perceptions of the environment or to reduce anxiety [19, 20]. However, most studies have been carried out with non-patients, were not randomized, were not true-to-life, and/or contained small sample groups [21]. Even though scientific evidence thus suggests a positive influence on people’s health, EBD can benefit from true-to-life rigorous studies with larger groups of patients.

An important indicator of health is stress [22–24]. Although patient’s recovery benefits from relaxation, this is often difficult because hospitalization is usually an uncertain and anxious experience [25]. Hospital environments can support these patients with anxiety mitigation. In this context, the theory of supportive design suggests that patients may experience less anxiety when the healthcare environment fosters more sense of control, access to social support, and access to positive distraction [26]. It is also known that the visual aesthetics can play an important role in mitigating anxiety in hospital facilities [27–29]. A complement of patient anxiety with their experienced sense of control, social support, positive distraction, and pleasantness of the room is thus advised.

The aim of this online study was to compare anxiety in an existing single-bed inpatient hospital room with three redesigns of this room in accordance with the principles of GR, FS, and EBD.

## Methods

### Participants

**Fig 1:**
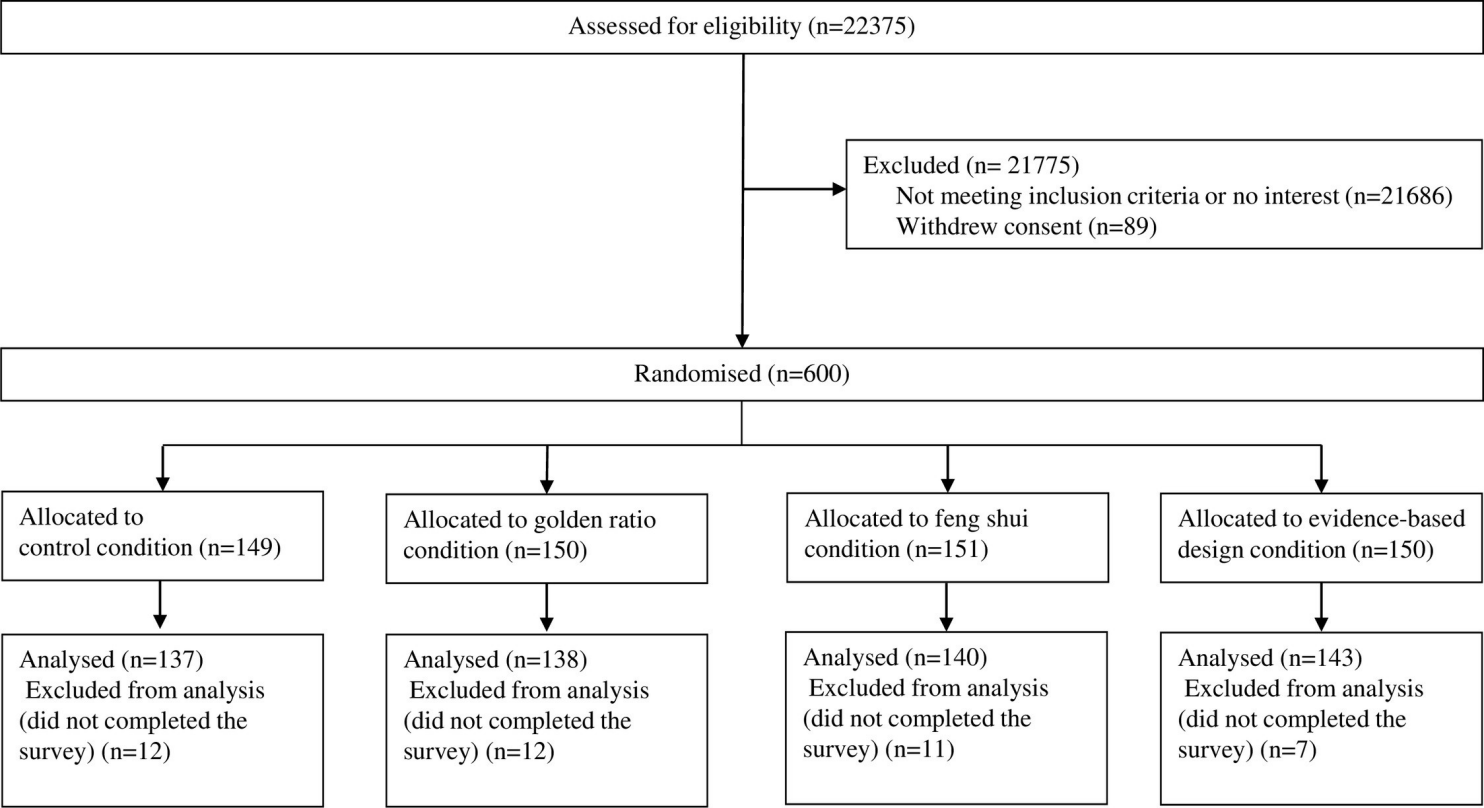
CONSORT flow diagram.

Participants were recruited between June 24, 2022, and August 22, 2022, by the Dutch Patient Federation (Fig 1). Eligible participants were patients of 18 years or older that had been hospitalized in the Netherlands in their lives for at least one night. Exclusion criteria were participants who were hospitalized for the last time: (1) at a psychiatric ward, (2) at a rehabilitation clinic, (3) or for the birth of their child, because this type of health care is often delivered at other facilities in the Netherlands and the length of stay differs too much from the average length of stay in a Dutch hospital of 4.5 days. Participants gave their written informed consent before continuing to the survey.

### Study design

The study was an online multi-arm parallel-group randomized controlled trial with participants assigned to one of four conditions, namely GR condition, FS condition, EBD condition, or the control condition (the digital twin of a real-life hospital room). Participants were exposed to a 3D simulation of the hospital room through a combination of video, images, and descriptions of the hospital room.

**Fig 2:**
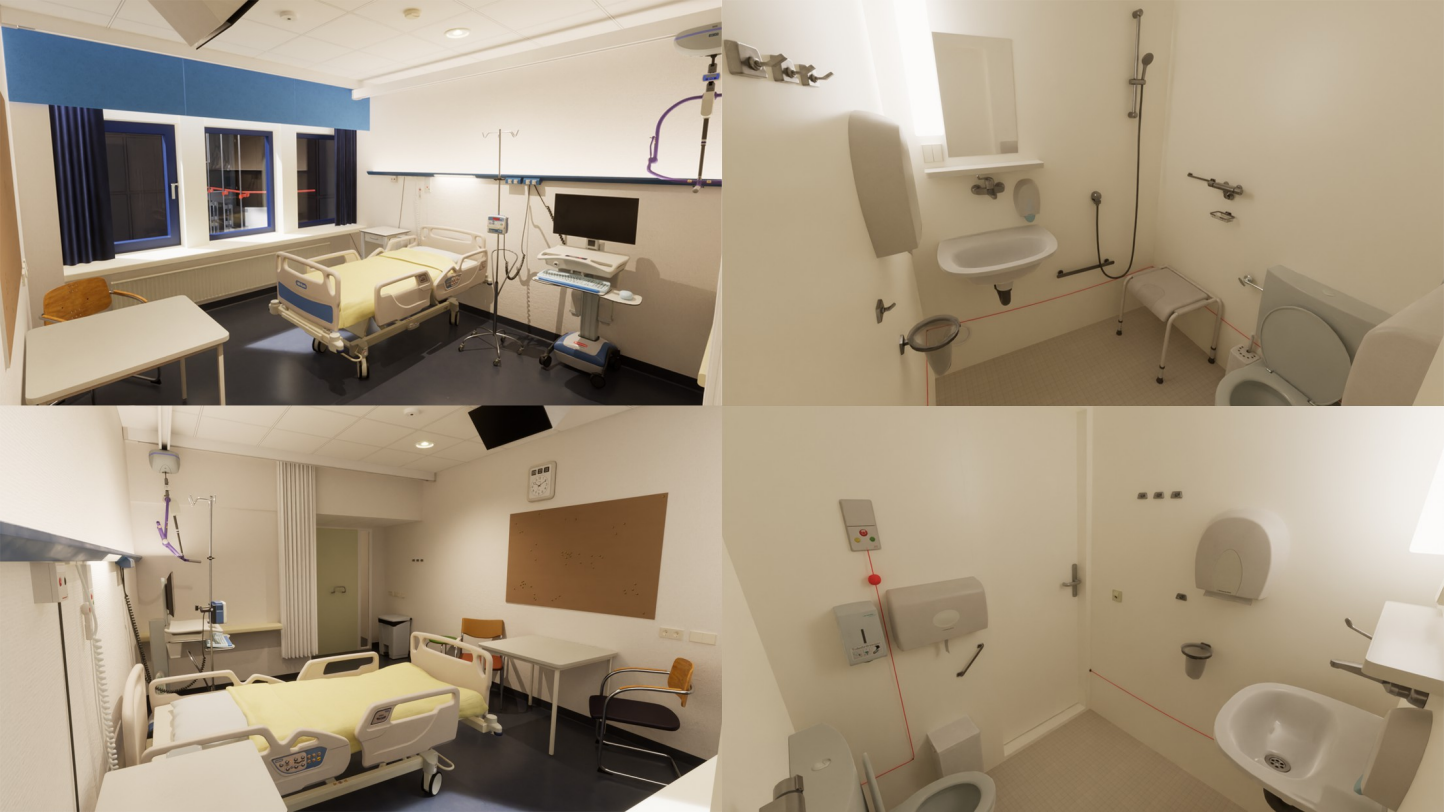
Images of the control condition.

In the *control condition* respondents experienced and rated the digital twin of a real-life single-bed inpatient hospital room with ensuite bathroom in an existing hospital in the Netherlands (see images Fig 2).

**Fig 3:**
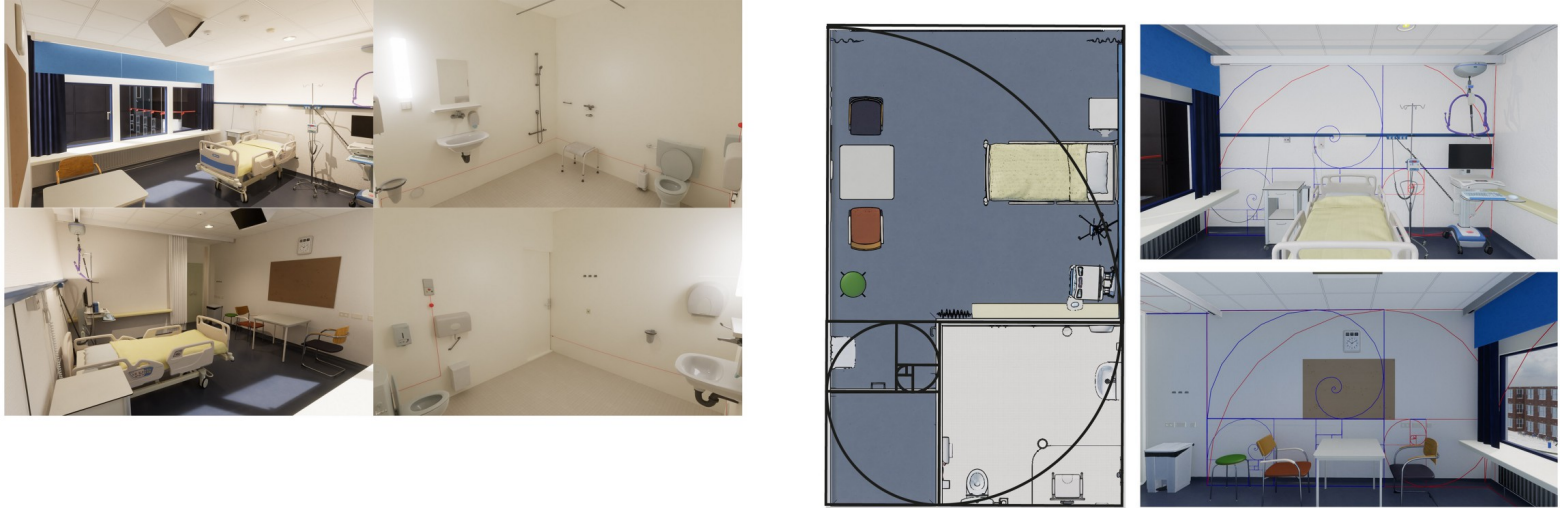
**a.** Images of the Golden Ratio condition. **b.** Alterations of specific design features in the Golden Ratio condition. All spatial rectangles of the inpatient hospital room and ensuite bathroom were shaped according to the GR of 1.618… The interior features in the room were horizontally positioned in the centre of the golden rectangles.

In the *GR condition* all spatial rectangles of the inpatient hospital room and ensuite bathroom were shaped according to the GR of 1.618… (see images Fig 3A and 3B). As the height of the ceiling (i.e., 2.83m) was subject to legal requirements, only this had to remain unchanged and formed therefore the basis for the design. For example, the height of the patient room was 2.83m so the width of the room was 4.58m (2.83m * 1.618…m). The interior features in the room were horizontally positioned in the centre of the golden rectangles. The height of the interior features remained unchanged because of ergonomic requirements.

**Fig 4:**
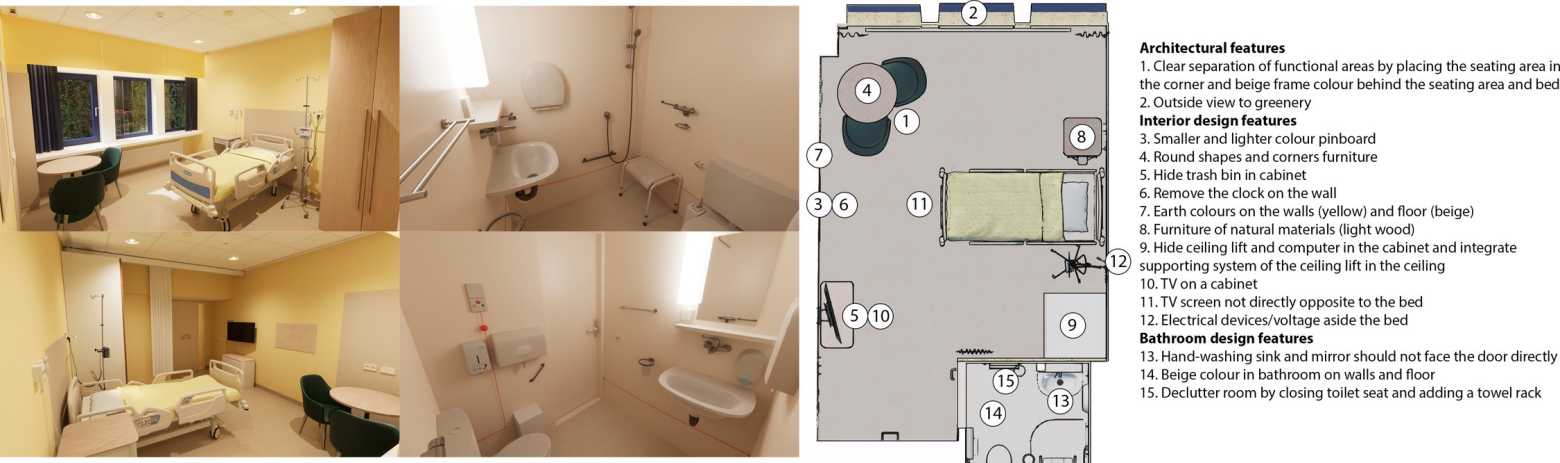
**a.** Images of the Feng Shui condition. **b.** Alterations of specific design features in the Feng Shui condition.

In the *FS condition* the interior features were designed according to the principles of Feng Shui (see images Fig 4A and 4B). This study focused on the FS Form School and the theory of five elements. Other FS related aspects (e.g., energy of flying stars, fortune of the patients and doctors, timing of usage, and Feng Shui of the entire hospital building) were excluded from this study due to the virtual study setting. For this study, a qualified FS master had determined the required design elements according to the FS principles. The first design intervention of the FS Form School is related to ‘position’ (e.g., supportive structure of colour block at the back of the bed), the second to ‘moderation of Qi flow’ (e.g., cabinet under the TV), the third to ‘types of Sha Qi’ (e.g., round shapes and corners of furniture, position TV to avoid reflection, removed clock, smaller pin board, hide trashcan in cabinet, avoid overhead structures like the ceiling lift and headwall, greenery outside to block the view on traffic), and the fourth to ‘arrangement’ (e.g., separation of functional areas by position and colour). From the theory of five elements, the design intervention is related to the colour scheme (i.e., earthy colour beige and fresh colour yellow because these colours represent centeredness, calmness, and balance in a hospital room). The FS list of changed features was limited to 15 features for reasons of feasibility and comparability. The proposed redesign was discussed within a qualified Feng Shui (grand) masters panel and after seven modifying iterations the redesign was approved.

**Fig 5:**
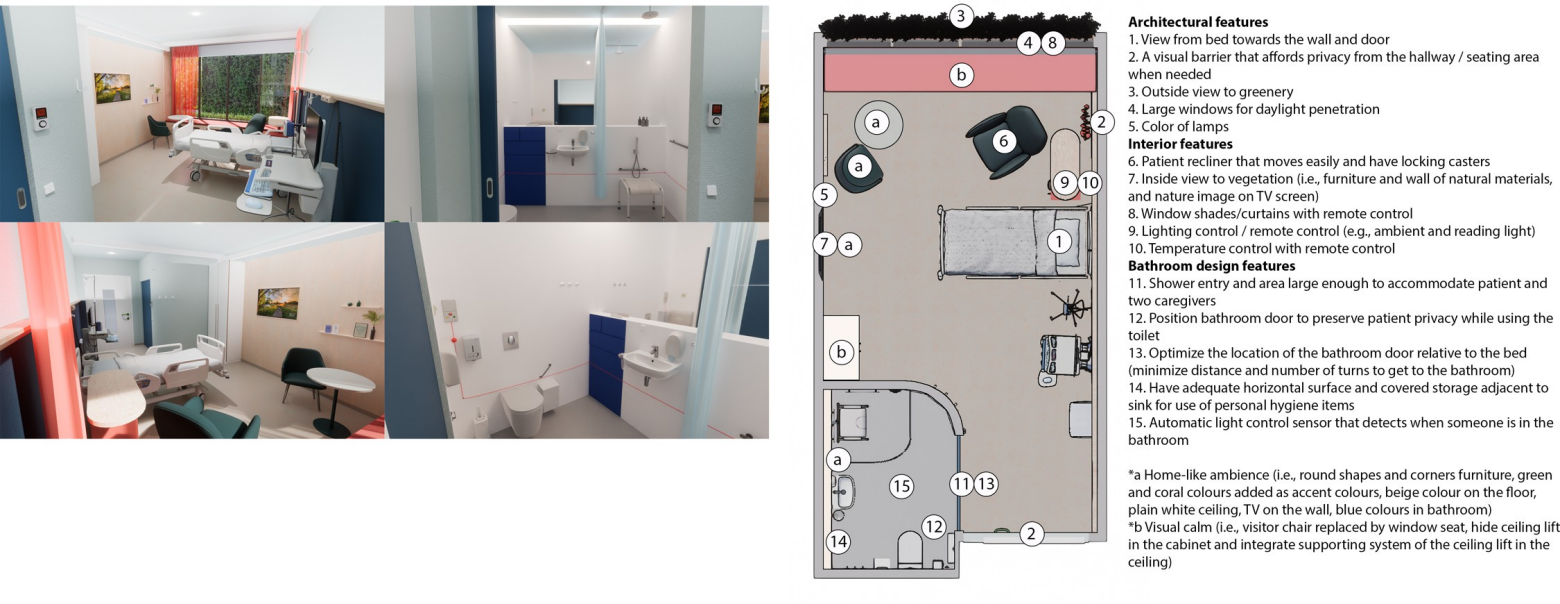
**a.** Images of the Evidence-Based Design condition, **b.** Alterations of specific design features in the Evidence-Based Design condition.

In the *EBD condition* the architectural and interior features were designed according to the principle of Evidence-Based Design (see images Fig 5A and 5B). The ambient features (i.e., sound and odour) were excluded from this study due to the focus on the visual environment and virtual study setting. In the first step, a longlist of 49 design features of single-bed inpatient rooms was identified based on two recent studies [15, 30]. In the second step, this list was assessed by a panel of scientific experts in this field using an online survey. They were asked to rate each feature on a 7-point scale how much impact they expect the feature would have on reducing anxiety (1 = no impact at all, 7 = a lot of impact). This was used to prioritize all features of the longlist. In the third step, the outcomes of the survey were discussed in a focus group with the same experts, to reach consensus about a shortlist of 15 design features. As a result, two design requirements were added, namely (1) a home-like ambience (e.g., use of materials, colours, shapes, lighting, and reduce view to medical devices), and (2) a visual calm (e.g., no chaos of ambience, styles, and elements). Finally, a leading architectural firm from London used the short list as a basis for the single-bed inpatient hospital room redesign of this study.

An overview of the design characteristics of the interventions of the four conditions is presented in the supporting information (S1 Table).

According to the Dutch Law for medical research involving human subjects (WMO), a waiver for ethical assessment was provided by the Medical Ethical Committee of the Medical University of Groningen on May 9, 2022 (METc 2022/259). The study was conducted according to the declaration of Helsinki.

### Randomisation

This study was an online double-blinded randomized controlled trial using the survey program Qualtrics. After some general questions about themselves they were automatically randomized by the online survey program to one of the four intervention groups (1:1:1:1 allocation ratio). Participants were told that the aim of this study was to improve hospital rooms for patients and that we were interested in their experiences and opinions about a hospital room. Participants were not aware of the different conditions. All outcome measures were self-reported by participants via an online survey (S1 Questionnaire).

### Procedures

After some general questions about themselves participants were randomized by the online survey program to one of the four intervention groups. First, they were exposed to a 2-minute video of the patient room that met one of the four conditions. In all conditions, participants were exposed to these videos which all followed the exact same ‘walking route’ through the room. Followed by two pictures of the patient room, two pictures of the bathroom, the 3D floorplan, and a detailed description of the facilities in the rooms. Participants were asked to imagine that they were recovering from surgery in this hospital room and then answered the questions about the dependent variable anxiety (STAI). As a reminder, they were again exposed to two pictures of the patient room and two pictures of the bathroom before answering the questions about the mediating variables (pleasantness of the room, sense of control, social support, and positive distraction).

### Outcomes

The primary outcome is anxiety. Anxiety was assessed by the short State-Trait Anxiety Inventory [31]. This six-item short form (STAI-6) measured the level of anxiety immediately after respondents had seen the intervention (i.e., calm, tense, upset, relaxed, content, and worried). Each item was measured from 1 (not at all) to 4 (very much). The positive items were reversed, and the sum of all items (total score of 6 to 24) was calculated. A higher score reflects more anxiety. There were no missing values on any of the self-reported items, because the online survey required an answer to all questions. Cronbach’s alpha was 0.85.

The measure to assess the mediators sense of control, social support, and positive distraction was the Supportive Hospital Environment Design Scale [32, 33]. The sense of control scale (5 items) measured the expected sense of control in the patient room. Each item was measured from (1) ‘strongly disagree’ to (5) ‘strongly agree’ (e.g., ‘In this hospital room I am able to control the surrounding environment’). A higher score reflects a more expected sense of control. The total score was 5 to 25. Cronbach’s alpha was 0.86. This social support scale (4-items) measured the expected social support in the patient room. Each item was measured from (1) ‘strongly disagree’ to (5) ‘strongly agree’ (e.g., ‘This hospital room allows me to socialize/“get together” with visiting family and friends’). A higher score reflects more expected social support. The total score was 4 to 20. Cronbach’s alpha was 0.87. This positive distraction scale (4-items) measured the expected perceived positive distraction in the patient room. Each item was measured from (1) ‘strongly disagree’ to (5) ‘strongly agree’ (e.g., ‘In this room my attention is drawn to interesting things’). A higher score reflects more expected positive distraction. The total score was 4 to 20. Cronbach’s alpha was 0.89.

The mediator pleasantness of the room (1 item) was assessed on a 10-point bipolar scale ranging from (1) ‘not pleasant’ to (1) ‘very pleasant’ directly after seeing the images for the second time of the patient room.

### Statistical analysis

In a priori power analysis a minimal sample size was calculated conservatively for the primary outcome anxiety and showed that we needed to include 70 participants in each group (total N = 280) to detect at least a medium effect size of *f* = 0.25 (alpha = 0.05, 1-beta = 0.95) in an ANOVA test with four groups. The sample size was calculated using the simplest between-group comparison (F tests–analysis of variance) in G*Power 3.1 [34, 35]. To remain 70 participants in each group, it was calculated that we needed to include 175 participants in each group (700 participants in total) with an estimated dropout of 60% (420/700). The total baseline of 700 participants can be taken as a conservative upper bound.

A linear regression analyses was conducted to test the effects of the intervention on perceived anxiety. Each intervention was compared with the control condition independently. The following possible explanatory variables were included in the model: gender, age, ethnicity, education, work, household, general health, and mental health. Next, the Akaike information Criterion (AIC) was used to identify variables significantly contributing to reduce anxiety [36]. The model selection was performed in R.

It was expected that participants would perceive less anxiety when they perceive the room as more pleasant, and, at the same time, that they would perceive more sense of control, more social support, and more positive distraction in the room. A parallel multiple mediation analysis was conducted to test for the indirect effect of the intervention on anxiety through social support and pleasantness of the room. Selection of potential mediators was based on the results of the minimum AIC model selection. We tested for the effect of the intervention, pleasantness of the room, social support, educational level, general health, and mental health. The mediation effect was estimated using PROCESS in SPSS 28.0 and performing 5000 bootstrap samples. The trial was registered retrospectively because of an oversight, prospective trial registration was overlooked (ISRCTN10480033). Details of our initial ethics approval and protocol are in the supported information.

### Role of the funding source

The funders had no role in study design, data collection and analysis, decision to publish, or preparation of the manuscript.

## Results

Participants were recruited between June 24, 2022, and August 22, 2022. During this study, 740 participants met the inclusion criteria of which 558 completed the questionnaire. From this group, 137 participants filled in the questionnaire in the control condition, 138 participants filled in the questionnaire in the GR condition, 140 participants filled in the questionnaire in the FS condition, and 143 participants filled in the questionnaire in the EBD condition (Fig 1). An ANOVA test (ratio variables) and chi-square test (nominal variables) were used to explore for differences between groups. No significant differences were found between the groups. The baseline characteristics of participants are presented in Table 1.

**Table 1:** Baseline characteristics for each group.

	Control condition	Golden Ratio condition	Feng Shui condition	Evidence-Based Design condition	*p*
	(n = 137)	(n = 138)	(n = 140)	(n = 143)	
Gender					0.493^a^
Male	68 (50%)	68 (49%)	79 (56%)	70 (49%)	
Female	69 (50%)	67 (49%)	60 (43%)	71 (50%)	
Other	0 (0%)	3 (2%)	1 (1%)	2 (1%)	
Age (mean, SD)	67.9 (9.4)	66.9 (11.0)	66.8 (8.9)	66.4 (9.3)	0.586^b^
Ethnicity					0.738^a^
Dutch	132 (96%)	132 (96%)	131 (94%)	136 (95%)	
Other	5 (4%)	6 (4%)	9 (6%)	7 (5%)	
Education					0.602^a^
Low	1 (1%)	1 (1%)	0 (0%)	0 (0%)	
Middle	60 (44%)	71 (51%)	62 (44%)	71 (50%)	
High	76 (55%)	66 (48%)	78 (56%)	72 (50%)	
Work					0.750^a^
Yes	18 (13%)	21 (15%)	25 (18%)	23 (16%)	
No	119 (87%)	117 (85%)	115 (82%)	120 (84%)	
Household					0.637^a^
Single	34 (25%)	33 (24%)	29 (21%)	36 (25%)	
Family	96 (70%)	94 (68%)	105 (75%)	95 (66%)	
Other	7 (5%)	11 (8%)	6 (4%)	12 (8%)	
General health (mean, SD)	2.6 (0.8)	2.6 (0.8)	2.6 (0.9)	2.7 (0.9)	0.994^b^
Mental health (mean, SD)	3.7 (1.0)	3.5 (1.0)	3.6 (0.9)	3.5 (1.0)	0.483^b^

^a^ Results Chi-Square test

^b^ Results ANOVA test

Regarding the primary measures, the EBD condition was associated with a significant reduction in anxiety and significant increases in sense of control, social support, positive distraction, and pleasantness of the room (all p<0.001; Table 2). The FS condition was associated with significant increases in social support, positive distraction, and pleasantness of the room (Table 2). Results showed no significant effects of the GR intervention on anxiety, pleasantness of the room, sense of control, social support, or positive distraction (Table 2).

**Table 2:** Primary outcome and mediators.

	Control condition	Golden Ratio condition	Feng Shui condition	Evidence-Based Design condition
	(n = 137)	(n = 138)	(n = 140)	(n = 143)
**Primary outcome**				
Mean anxiety (SD)	10.26 (3.57)	10.62 (3.65)	9.61 (3.30)	8.90 (3.08)
Change in mean from baseline (95% CI)	-	0.37 (-0.44 to 1.17)	-0.65 (-1.45 to 0.15)	-1.35 (-2.15 to -0.55)
Cohen’s d	-	0.11	-0.19	-0.40
p value	-	0.371	0.113	<0.001
**Mediators**				
Mean sense of control (SD)	15.09 (4.52)	14.86 (5.14)	15.56 (4.78)	18.27 (5.09)
Change in mean from baseline (95% CI)	-	-0.24 (-1.40 to 0.92)	0.47 (-0.69 to 1.62)	3.17 (2.02 to 4.32)
Cohen’s d	-	-0.05	0.10	0.65
p value	-	0.684	0.425	<0.001
Mean social support (SD)	16.03 (3.72)	15.45 (4.06)	17.31 (2.81)	18.04 (2.80)
Change in mean from baseline (95% CI)	-	-0.58 (-1.38 to 0.22)	1.29 (0.49 to 2.08)	2.01 (1.22 to 2.81)
Cohen’s d	-	-0.17	0.38	0.60
p value	-	0.156	0.002	<0.001
Mean positive distraction (SD)	10.16 (4.23)	10.85 (4.49)	12.54 (4.19)	14.85 (4.15)
Change in mean from baseline (95% CI)	-	0.69 (-0.32 to 1.70)	2.38 (1.37 to 3.38)	4.69 (3.69 to 5.69)
Cohen’s d	-	0.16	0.56	1.10
p value	-	0.182	<0.001	<0.001
Mean pleasantness of the room (SD)	6.81 (2.36)	6.86 (2.39)	7.88 (1.79)	8.42 (1.73)
Change in mean from baseline (95% CI)	-	0.45 (-0.45 to 0.54)	1.07 (0.58 to 1.56)	1.61 (1.12 to 2.10)
Cohen’s d	-	0.02	0.51	0.77
p value	-	0.858	<0.001	<0.001

**Fig 6:**
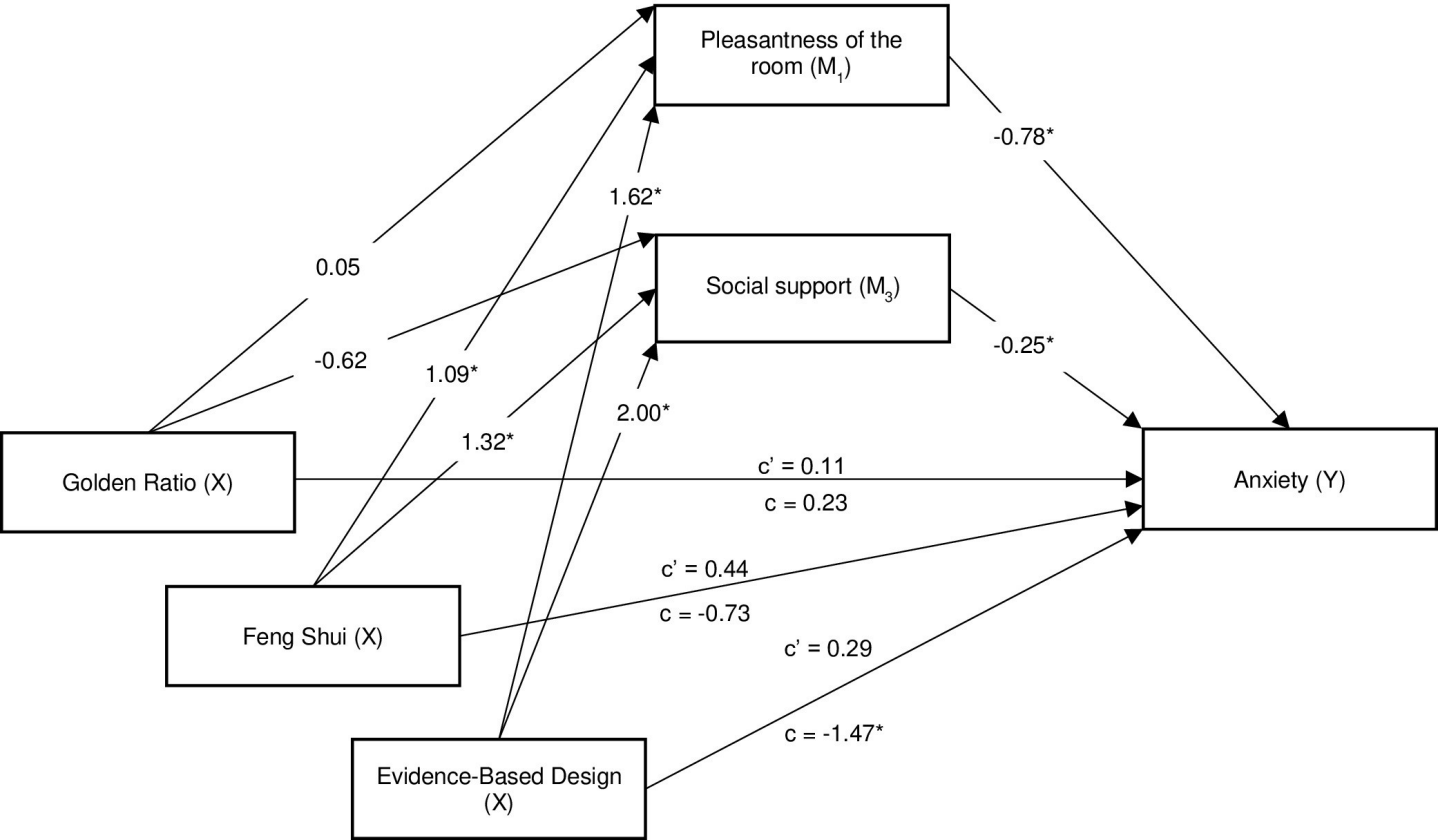
Unstandardized coefficients (B) of parallel multiple mediation analysis with direct effects, total effects, and indirect effects of the design interventions on anxiety though pleasantness of the room and social support (see Table 4). *p < 0.01; c’ = direct effect; c = total effect.

Table 3 shows the results of the linear regression analyses. Although results showed no direct effect of the intervention on anxiety, results of the parallel multiple mediation analysis in Table 4 showed a significant total effect of the EBD intervention on anxiety, as the 95% bootstrapped confidence interval did not include zero (B = -1.47, CI = -2.25 to -0.68). Results also showed a significant indirect effect of the intervention EBD on anxiety through the pleasantness of the room (B = -1.26, CI = -1.70 to -0.85) and through social support (B = -0.50, CI = -0.77 to -0.27). As Fig 6 illustrates, the group of participants who were exposed to the EBD condition rated the room as more pleasant (B = 1.62, *p* = <0.001) and expected more social support (B = 2.00, p = <0.001). The more pleasant participants perceived the room, the less anxiety they reported (ab = 1.62*-0.78 = 1.26). Moreover, the more social support participants expected, the less anxiety they reported (ab = 2.00*-0.25 = -0.50). No evidence was found for the mediating effects of either sense of control or positive distraction on anxiety.

**Table 3:** Results linear regression analyses on anxiety.

	Anxiety			Anxiety (after min. AIC)^b^		
	Coef.	SE	p	Coef.	SE	p
(intercept)	20.13	1.01	<0.001	21.00	0.65	<0.001
Control condition^a^						
Golden Ratio condition	0.10	0.30	0.734			
Feng Shui condition	0.44	0.31	0.154			
Evidence-Based Design condition	0.31	0.32	0.335			
Pleasantness of the room	-0.78	0.07	<0.001	-0.75	0.07	<0.001
Sense of control	-0.01	0.03	0.770			
Social support	-0.25	0.05	<0.001	-0.25	0.04	<0.001
Positive distraction	0.01	0.04	0.734			
Female^a^						
Male	0.02	0.23	0.931			
Other	0.41	1.03	0.693			
Age	0.02	0.01	0.236			
High education^a^						
Middle education	0.53	0.22	0.016	0.51	0.21	0.016
Low education	5.91	1.77	<0.001	5.92	1.74	<0.001
Household; family^a^						
Household; single	-0.30	0.26	0.236			
Household; other	-0.16	0.44	0.713			
Work; Yes	0.01	0.33	0.968			
Ethnicity; Other	0.07	0.50	0.893			
General health	0.18	0.14	0.194	0.20	0.14	0.145
Mental health	-0.64	0.12	<0.001	-0.60	0.12	<0.001

^a^ Marks as reference category

^b^ Results with the minimum Akaike Information Criterion (AIC) for model selection

**Table 4:** Results parallel multiple mediation analysis. Coef. = coefficient, SE = Standard error.

	Anxiety			Pleasantness of the room			Social support			Anxiety (total effect)		
	Coef.	SE	p	Coef.	SE	p	Coef.	SE	p	Coef.	SE	p
(intercept)	22.78	0.88	<0.001	6.94	0.56	<0.001	18.38	0.90	<0.001	12.81	0.90	<0.001
Golden Ratio condition	0.11	0.30	0.712	0.05	0.25	0.843	-0.62	0.40	0.125	0.23	0.40	0.576
Feng Shui condition	0.44	0.30	0.142	1.09	0.25	<0.001	1.32	0.10	0.001	-0.73	0.40	0.068
Evidence-Based Design condition	0.29	0.31	0.345	1.62	0.25	<0.001	2.00	0.40	<0.001	-1.47	0.40	<0.001
Pleasantness of the room	-0.78	0.04	<0.001									
Social support	-0.25	0.07	<0.001									
Education	-0.64	0.21	0.003	-0.30	0.18	0.086	-1.10	0.28	<0.001	-0.13	0.28	0.645
General health	0.19	0.14	0.179	0.03	0.12	0.814	-0.18	0.19	0.341	0.21	0.19	0.265
Mental health	-0.58	0.12	<0.001	0.15	0.10	0.129	0.25	0.16	0.125	-0.76	0.16	<0.001

Next, results also showed a significant indirect effect of the FS intervention on anxiety through the pleasantness of the room (B = -0.85, CI = -1.29 to -0.45) and through social support (B = -0.33, CI = -0.57 to -0.13). As Fig 6 illustrates, the group of participants who were exposed to the FS condition rated the room as more pleasant (B = 1.09, *p* = <0.001) and expected more social support (B = 1.32, p = 0.001). The more pleasant participants perceived the room, the less anxiety they reported (ab = 1.09*-0.78 = -0.85). Moreover, the more social support participants expected, the less anxiety they reported (ab = 1.32*-0.25 = -0.33). No evidence was found for the mediating effect of sense of control and positive distraction on anxiety.

## Discussion

In this online study, we developed three single-bed inpatient hospital room redesigns and found evidence for the effects of the FS and EBD principles on anxiety with the original room design as a baseline. In contrast, there was no evidence to support a GR application as it did not influence any of the outcomes.

As expected, participants in the online EBD condition perceived less anxiety compared to the control condition. Results showed statistically significant evidence for the total effect of the EBD condition on anxiety. Moreover, this study also showed that the anxiety-inducing effect of the EBD condition is mediated by perceived pleasantness of the room and social support. Participants perceived the room as pleasant and expected more social support in the EBD condition, which in turn reduced their reported anxiety levels. This partially confirms the theory of supportive design that perceived social support can reduce anxiety [26], but also suggests that other mediating variables may have influenced reported anxiety levels. However, results also showed that even though the EBD condition significantly improved sense of control and positive distraction, sense of control and positive distraction both failed to influence the anxiety outcome. This can be explained by the fact that the people in our sample were relatively old, with an average age of 66 years. This may imply that not all people perceive higher levels of control as anxiety reducing. It is possible that the mere presence of functionalities and technologies can stress out people. In this context, for elderly it is important that technologies are accessible ‐ that information about the technology is available ‐ and that it is easy to use [37]. If such needs are ignored, it may provoke anxiety. Moreover, elderly can be frightened to learn new things, such as how to handle new technologies [38]. The results also imply that a pleasant looking room is more important for anxiety reduction than offering positive distraction. As pleasantness of the room is mostly positive associated with reducing anxiety [27]. Some evidence exists in literature, but further research should clarify which specific design features positively influence anxiety best.

In the online FS condition a significant indirect effect was shown on anxiety, through the rating of pleasantness of the room and expected social support. Participants experienced the room as pleasant and expected to receive more social support in the FS condition. Results showed significant mediation effects, but lacked an overall effect of FS on anxiety. The most likely interpretation of these results is that FS has a stronger influence on the outcomes perceived pleasantness and social support, when compared with the anxiety outcome [39]. Another possible explanation might be that the FS intervention has an additional negative effect on an unmeasured mediator which leads to an anxiety increase [39–41]. This might be due to the unmeasured aspect of functionality. For example, in the open questions participants in the FS condition stated that they disliked the position of the TV, because they always had to look to the side, whereas others argued that they missed storage space for personal belongings. This may imply that as the position of the TV did not “work” appropriately, it may have created adverse effects to anxiety reduction [29]. Further research in a real-life setting should clarify which other FS aspects can mitigate anxiety best. Nevertheless, in the FS condition participants experienced a more pleasant looking room and more social support, and accordingly experienced less anxiety. Thus, it seems that the energetic FS condition, just as EBD, reduces anxiety.

The online GR condition showed no effects on anxiety, perceived pleasantness of the room, sense of control, social support, and positive distraction. The first explanation might be that due to the new proportions and positioning, more attention is given to the existing, apparently, unpleasant design features as reported in some of the open questions. For example, in the open questions participants in the GR condition stated that they disliked the colours on the floor and walls, and the clinical atmosphere. Secondly, it can perhaps also be explained with spatial disproportionality, in this case a room size that is too large for the existing interior features [29]. This suggests that the design of a patient room cannot be improved by mechanical application of the GR design principle; perhaps a more refined application of GR is better fit for purpose. Nevertheless, it can also be that a combination of GR with EBD or FS may have a stronger influence on anxiety through pleasantness of the room and social support. Further research should clarify this.

## Limitations and further research

There are some limitations to be considered. In the current study, multiple design features were manipulated simultaneously. Although this study showed that both FS and EBD are capable in effecting anxiety, further research should continue to study the effect of specific design features. Another limitation is that only single-bed inpatient rooms were studied. Nowadays, hospitals in Europe and United States are designed with an increasing number of single rooms. Nevertheless, from a social perspective multi-bedded rooms should still be considered [42]. Therefore, it is recommended that further studies also study the effects of these design principles in multi-bedded inpatient rooms.

Although this study showed that simulation is a useful tool to study the effects of different design, it also has some limitations. First, participants were not exposed to real hospital rooms during hospitalization. Further research in real hospital settings should assess the effects of the actual health of participants and health care delivery. Visual simulations of an inpatient room are likely to have limitations for assessing the effects of design features that enhance control. Decades of research have found that sense of control is important for reducing anxiety [26, 43, 44]. These studies have commonly assigned participants to real or tangible multisensory environmental or laboratory conditions that vary with respect to the actual experience of being able to control. For example, visual simulations do not give participants an actual experience of being able to control, for instance, lighting or temperature or watching a program on the TV, or privacy violations. Second, a limitation of this study is that we did not include the audio environment in the visual simulation. Much EBD theory supports the importance of noise-reducing design principles for reducing patient stress and improving clinical outcomes. Further research in a more realistic and valid simulation would require more advanced digital technology or interactive audio-visual virtual reality. Third, another limitation of this study was that only FS form school could be included in this simulation study. Some major aspects of FS, for instance, energy of flying stars, fortune of the patients and doctors, timing of usage, and FS of the entire hospital, were not included in this simulation study. Further research in a real hospital environment could include these different FS schools. Notwithstanding these limitations, this current simulation study contributes to a better understanding of the influence of different design principles on anxiety. Understanding the influence of these design principles will contribute to the further development of effective interventions in inpatient room designs.

## Conclusions

In summary, participants in the online FS and EBD conditions experienced less anxiety, through experiencing a more pleasant room and more social support. The design principle FS can be demystified, and our results suggest that there are many similarities between FS and EBD, even though they exist from totally different cultural backgrounds and led to different overall interior designs. A well-known example of similarity is the outside view to greenery [8, 10]. Other examples are the use of similar round shapes and corners in furniture [8], decluttering [8, 30], and the use of natural materials [15]. Knowledge about the effects of these design principles and related design features can help designers and decision makers to create healthcare building facilities for patients that have the capacity to reduce their anxiety, and, by doing so, can enhance their health. Our conclusion is that designing a patient room according to FS and EBD principles can create a more pleasant room to reduce anxiety.

## Supporting information

S1 Table Design features of interventions. (DOCX)

S1 Questionnaire (DOCX)

S1 Dataset (XLSX)

S1 Checklist CONSORT 2010 checklist of information to include when reporting a randomised trial*. (DOC)

S1 Protocol (PDF)
