# Supplementary material for: A randomized controlled trial of Golden Ratio, Feng Shui, and evidence based design in healthcare
Source: PLoS One. 2024 Jun 5;19(6):e0303032. doi: 10.1371/journal.pone.0303032 (PMC11152261; doi:10.1371/journal.pone.0303032)
Supplement: S1 Table — (DOCX) [file pone.0303032.s001.docx]

|  | **Control condition** | **Golden Ratio condition** | **Feng Shui condition** | **Evidence-Based Design condition** |
| --- | --- | --- | --- | --- |
| **Architectural features** | | | | |
| General layout |  | 1. Adjusted spatial proportions room | 1. Clear separation of functional areas by placing the seating area in the corner and beige frame colour behind the seating area and bed | 1. View from bed towards the wall and door |
|  |  |  |  | 2. A visual barrier that affords privacy from the hallway / seating area when needed |
| Windows |  | 2. Adjusted proportions windows | 2. Outside view to greenery | 3. Outside view to greenery |
|  |  |  |  | 4. Large windows for daylight penetration |
| Lighting |  |  |  | 5. Color of lamps |
| **Interior design features** | | | | |
| Furniture |  | 3. Position interior elements in the center of Golden Rectangles | 3. Smaller and lighter colour pinboard | 6. Patient recliner that moves easily and have locking casters |
|  |  |  | 4. Round shapes and corners furniture | *^1^ Round shapes and corners furniture |
|  |  |  |  |  |
|  |  |  | 5. Hide trash bin in cabinet | *^2^ Minimize furniture (i.e., vistor chair replaced by window seat). |
|  |  |  | 6. Remove the clock on the wall |  |
| Colour scheme |  |  | 7. Earth colours on the walls (yellow) and floor (beige) | *^1^ Green and coral colours added as accent colours. Beige colour on the floor. Plain white ceiling. |
| Nature inside |  |  | 8. Furniture of natural materials (light wood) | 7. Inside view to vegetation (i.e., furniture and wall of natural materials, and nature image on TV screen) |
| Curtains |  |  |  | 8. Window shades/curtains with remote control |
| Equipment |  |  | 9. Hide ceiling lift and computer in the cabinet and integrate supporting system of the ceiling lift in the ceiling | *^2^ Hide ceiling lift in the cabinet and integrate supporting system of the ceiling lift in the ceiling |
|  |  |  | 10. TV on a cabinet | *^1^ TV on the wall |
|  |  |  | 11. TV screen not directly opposite to the bed | 9. Lighting control / remote control (e.g., ambient and reading light) |
|  |  |  | 12. Electrical devices/voltage aside the bed | 10. Temperature control with remote control |
| **Bathroom design feature** | | | | |
| General layout |  | 4. Adjust spatial proportions bathroom | 13. Hand-washing sink and mirror should not face the door directly | 11. Shower entry and area large enough to accommodate patient and two caregivers |
|  |  |  |  | 12. Position bathroom door to preserve patient privacy while using the toilet |
|  |  |  |  | 13. Optimize the location of the bathroom door relative to the bed (minimize distance and number of turns to get to the bathroom) |
| Colour scheme |  |  | 14. Beige colour in bathroom on walls and floor | *^1^ Blue colours in bathroom |
| Furniture |  | 5. Position interior elements in the center of Golden Rectangles | 15. Declutter room by closing toilet seat and adding a towel rack | 14. Have adequate horizontal surface and covered storage adjacent to sink for use of personal hygiene items |
| Lighting |  |  |  | 15. Automatic light control sensor that detects when someone is in the bathroom |

*^1^ Home-like atmosphere

*^2^ Visual calm
