# Supplementary material for: A randomized controlled trial of Golden Ratio, Feng Shui, and evidence based design in healthcare
Source: PLoS One. 2024 Jun 5;19(6):e0303032. doi: 10.1371/journal.pone.0303032 (PMC11152261; doi:10.1371/journal.pone.0303032)
Supplement: S1 Questionnaire — (DOCX) [file pone.0303032.s002.docx]

# S1 Questionnaire

| Scale / dimension | Question | Answer type: |
| --- | --- | --- |
| Gender | 1. With which gender do you identify yourself? | Male, Female, Other |
| Age | 1. What is your age? | Years |
| Education | 1. What is your highest level of education? | Low, Middle, High |
| Household | 1. What is your household composition? | With partner, with child(ren), with partner and child(ren), without partner and child(ren) |
| Ethnicity | 1. Where were you born? | The Netherlands, Western Europe, Eastern Europe, Middle East, Asia, Africa, North America, Central America, South America,  Canada, Australia / New Zealand |
| Work | 1. Do you perform paid work? | Yes, NoDo |
| General health  (SF-12) | 1. In general, would you say your health is: | 1. Poor 2. Fair 3. Good 4. Very good 5. Excellent |
| Mental health  (SRMH) | 1. In general, would you say your mental health is: | 1. Poor 2. Fair 3. Good 4. Very good 5. Excellent |
| Anxiety  (STAI) | 1. I feel calm 2. I am tense 3. I feel upset 4. I am relaxed 5. I feel content 6. I am worried | 4-point Likert scale:   - Not at all (1) - Somewhat (2) - Moderately (3) - Very much (4) |
| Sense of control  (SHEDS) | 1. In this hospital room I am able to control the surrounding environment. 2. I can control the physical features and materials of my hospital room 3. There are choices I can make about the physical features of my hospital room. 4. In this room I can adjust, re-arrange, and re-organize things in my hospital room as needed. 5. I determine the organization/appearance of my hospital room. | 5-point Likert scale:   - Strongly disagree (1) - Disagree somewhat(2) - Neither agree or disagree (3) - Agree somewhat (4) - Strongly agree (5) |
| Social support  (SHEDS) | 1. This hospital room allows me to socialize/”get together” with visiting family and friends. 2. My family and friends would feel comfortable in this hospital room. 3. In this hospital room I could enjoy the company of visiting family and friends. 4. This hospital room provides a supportive environment for visiting family and friends. | 5-point Likert scale:   - Strongly disagree (1) - Disagree somewhat(2) - Neither agree or disagree (3) - Agree somewhat (4) - Strongly agree (5) |
| Positive distraction  (SHEDS) | 1. In this room my attention is drawn to interesting things. 2. In this room there are objects that attract my attention. 3. In this room I am absorbed by the surroundings. 4. There is plenty that I want to linger on here. | 5-point Likert scale:   - Strongly disagree (1) - Disagree somewhat(2) - Neither agree or disagree (3) - Agree somewhat (4) - Strongly agree (5) |
| Pleasantness of the room | 1. Please indicate how pleasant you find the room’s appearance? | 10-point bipolar scale  Not pleasant (1) vs. Very pleasant (10) |
