# Supplementary material for: A randomized controlled trial of Golden Ratio, Feng Shui, and evidence based design in healthcare
Source: PLoS One. 2024 Jun 5;19(6):e0303032. doi: 10.1371/journal.pone.0303032 (PMC11152261; doi:10.1371/journal.pone.0303032)
Supplement: S1 Protocol — (PDF) [file pone.0303032.s005.pdf]

The impact of the hospital inpatient room on patients' stress

Hospital Room of the Future; M2205 0157

(non-WMO study protocol)

Date of document: 04-05-2022
Version number: 1.0

CONTENT

1. STUDY ORGANIZATION 3
2. PROTOCOL SIGNATURE SHEET 4
3. ABSTRACT 5
4. BACKGROUND 5
5. METHOD 6
5.1 Description study design 6
5.2 Design 7
5.3 Population 7
5.4 Recruitment and informed consent/objection 8
5.5 Research Data Management Plan (RDMP) 9
5.6 Management of biomaterials 11
5.7 Burden, Risks & Benefits (Prospective studies only) 11
5.8 Incidental findings 12
5.9 Data analysis 12
5.10 Participant information after the study 12
5.11 Research revenue 12
6. REFERENCES 13
7. APPENDICES (if applicable) 14
Appendix I Informed Consent and Questionnaire 15
Appendix II Data Management Plan 25
Appendix III Statistical Analysis Plan 33

1. STUDY ORGANIZATION

**Study title**	The impact of the hospital inpatient room on patients' stress
**Planned start date**	15-05-2022
**Estimated completion date**	01-07-2023
**Project leader (external)**	Emma Zijlstra, Post doc Researcher, Hanze University of Applied Sciences, Research Group Facility Management
**(Principal) investigator UMCG**	NA
**Researcher(s) ~~UMCG~~**	Sjoukje van Dellen, Post Doc Researcher, Hanze University of Applied Sciences, Research Group Facility Management
**Corresponding researcher UMCG**	NA
**(Principal) investigator other centers**	Mark Mobach, Professor Facility Management, Hanze University of Applied Sciences, Research Group Facility Management
**Sponsor (in Dutch: verrichter/opdrachtgever)**	Hanze University of Applied Sciences
**Financial support/subsidising party**	NA
**Collaboration with non-profit Laboratory / research sites (in- and outside UMCG)**	NA
**Collaboration with commercial parties / companies (in- and outside UMCG)**	NA
**Name bio- or databank and bankmanager**	NA
**Name previous study ('FAIR data') and (principal) investigator**	NA

2. PROTOCOL SIGNATURE SHEET

The undersigned (Principal) investigator and head of department UMCG confirm that the study and its procedures will comply with the present study protocol and the nWMO Kaderreglement UMCG. Without ethical approval the data/biomaterials will not be used for other (research) purposes (e.g. 'FAIR data').

**Name**	**Signature**	**Date**
**(Principal) investigator:**		04-05-2022
	Mark Mobach, Professor Facility Management, Hanze University of Applied Sciences	
**Other (if applicable)**		04-05-2022
	Emma Zijlstra, Post doc Researcher, Hanze University of Applied Sciences	

3. ABSTRACT

- **Background**
  Many patients experience stress during hospitalization (Andrade & Devlin, 2015; Zijlstra, 2021). The theory of supportive design conceptualizes that the physical environment improves the sense of control, access to social support and positive distraction which positively effects patients' well-being (Ulrich, 1991).
- **Main research question**
  What is the effect of a single-bed inpatient room design on patients' stress?
- **Design (including population, confounders/outcomes)**
  The study is an online randomized controlled trial with a between-subjects design. Participants are assigned to one of four conditions, namely the current patient room, a patient room with architectural changes, a patient room with interior changes, a patient room with architectural and interior changes. Participants are included when they have been hospitalized in the last 5 years in a Dutch hospital. Perceived stress, sense of control, social support, positive distraction, pleasantness of the room, and environmental sensitivity are measured using a questionnaire.
- **Expected results**
  It is expected that participants exposed to changed design features anticipated less stress than participants that are exposed to the current patient room (control condition). It is expected that this positive effect is mediated by sense of control, positive distraction, social support, and perceived pleasantness. It is also expected that participants who score higher in environmental sensitivity perceive more sense of control, positive distraction, social support and perceived pleasantness in the new design and, therefore, perceive less stress.

4. BACKGROUND

- **Introduction and rationale**
  Many patients experience stress during hospitalization (Andrade & Devlin, 2015; Zijlstra, 2021). Patients are often concerned about their disease and recovery in inpatient settings. In these hospital settings, the impact of the design of inpatient rooms on patients is still not well understood. Knowledge regarding the influence of this environment on patients is essential for facilitating the quality of health care. But how to design patient rooms that actually improve patients' well-being? Therefore, is important to understand the holistic experience of patients in a patient room.
  Patient room design is receiving growing attention to lower hospital-acquired infection rates during hospitalization (Taylor et al., 2018). For that reason, hospitals are nowadays designed with an increased number of single rooms (Larsen et al., 2014). Although the building costs for Dutch hospitals are high and approximately around €3.000,- per square meter, building decisions are still mainly based on experience and intuition but not on scientific evidence (Becker & Parsons, 2007).
  The theory of supportive design conceptualizes that the physical environment improves the sense of control, access to social support and positive distraction which positively effects patients' well-being (Ulrich, 1991). Several studies have shown the advantages of single patient room elements. These studies showed that, for example, plants, view on nature, daylight, and opportunity for social support can positively affect patients physical and psychological well-being (Dijkstra, 2009; Suess & Mody, 2018; Ulrich et al., 2004). However, until now there have been no

empirical tests of the effects of a holistic single inpatient room design on patients' well-being. The aim of this study is to test whether design principles of a holistic inpatient room has stress reducing effects, because they improve the perceptions of control, social support, positive distraction, and perceived pleasantness of the room.

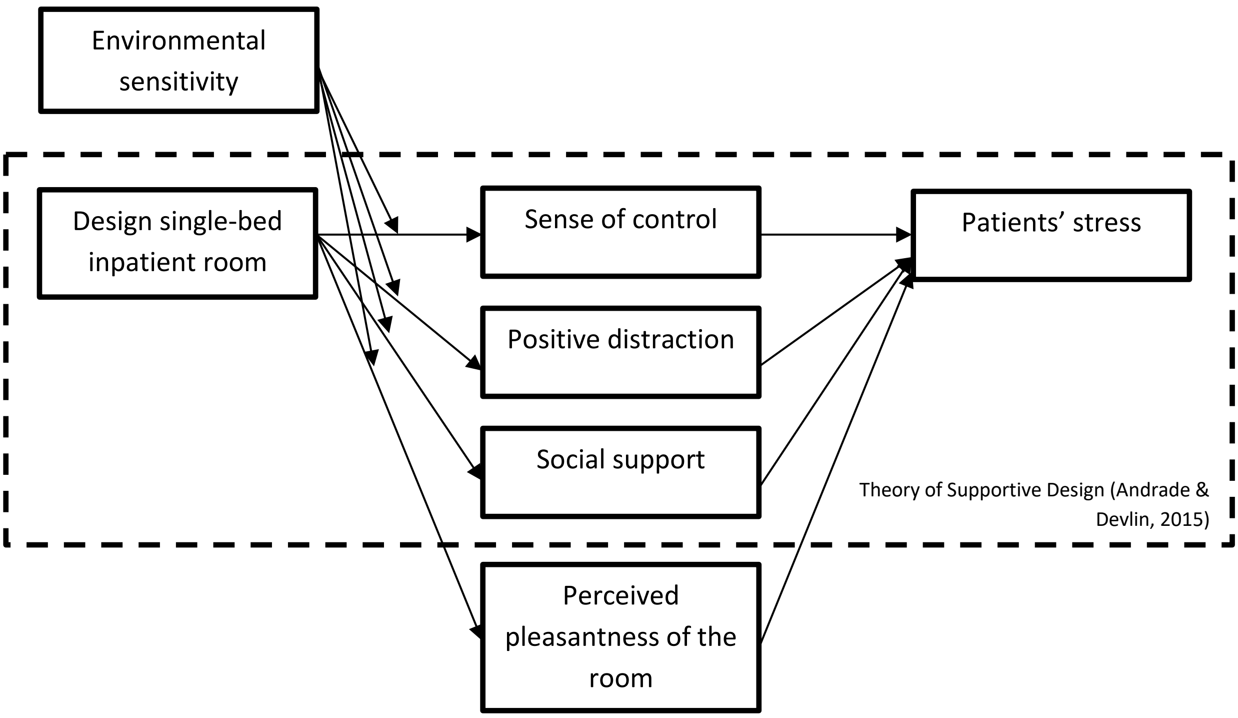

- **Research question** (**Bank or FAIR data**)
  What is the effect of a single-bed inpatient room design on patients' stress?

5. METHOD

5.1 Description study design

The study is an online randomized controlled trial with a between-subjects design. Participants are assigned to one of four conditions, namely (1) the control condition which is the current UMCG patient room, (2) a patient room with architectural changes, (3) a patient room with interior changes, (4) a patient room with architectural and interior changes.

It is expected that participants exposed to a new design anticipated less stress than participants that are exposed to the current patient room. It is expected that the positive effect of the design is mediated by sense of control, positive distraction, social support, and perceived pleasantness. Participants perceive more sense of control, positive distraction, social support, and pleasantness of the room, and, therefore, perceive less stress. It is also expected that participants who score higher in environmental sensitivity perceive more sense of control, positive distraction, social support and pleasantness in a new design and, therefore, perceive less stress compared to the control condition.

To answer the main question participants will be asked to complete a questionnaire that will take approximately 20 minutes to complete (see Appendices).

In collaboration with the Dutch Patient Federation participants will be selected and approached to ask if they wish to participate in this study. When participants are willing to participate in this study they will receive an informed consent and online questionnaire. This questionnaire contains photographs and a video of the patient room and contains general questions about the person, questions about their perception of the room and questions about the psychological well-being.

5.2 Design

5.2.1 Mono- or multicenter study	Mono-center study<br>no	Multicenter study<br>yes
This study is performed in the Netherlands. Participants are recruited via the Dutch Patient Federation. Eligible participants are of this study are patients who were hospitalized in a hospital in the Netherlands the last 5 years.		
5.2.2 Retrospective study (available data/ biomaterials only) or prospective study (data/ biomaterials from [some] participants will be collected in the future).	Retrospective study<br>no<br>**skip**<br>**Sections 5.4.2/5.5.6**	Prospective study<br>yes<br>**skip**<br>**Sections 5.4.1**
Participants will be asked to virtually experience a hospital room in an online questionnaire.		
5.2.3 Cross-sectional or follow-up study	Cross-sectional study<br>yes	Follow-up study<br>no
5.2.4 Quantitative or qualitative study (click both if mixed-method)	Quantitative study<br>yes	Qualitative study<br>yes
Participants are asked to answer closed and open questions in a questionnaire (quantitative and qualitative data)		
5.2.4 Pilot study	no	

5.3 Population

5.3.1 Inclusion and exclusion criteria	
• Inclusion criteria: 18 years or older, have been hospitalized for at least 1 night in the last 5 years in the Netherlands, sufficient knowledge of the Dutch language<br>• Exclusion criteria: have been hospitalized at a psychiatric ward or IC unit, have been hospitalized in a revalidation clinic, have been hospitalized for a birth	
5.3.2 Number of participants	
• Target total number of participants: 834<br>• Target number of UMCG participants: NA	
5.3.3 Study subjects (tick all that apply)	
• Healthy volunteers<br>• Patients	yes<br>no
Participants are volunteers of a patient panel of the Dutch Patient Federation.	
5.3.4 Subject classification (tick all that apply)	
• Participants ≥ 16 years<br>• Children between 12 and 16 years *(if applicable, written informed consent will be obtained from child and both parents - if both have authority, or guardian [or parents/guardian only if incapacitated child])*<br>• Children < 12 years *(if applicable, written informed consent will be obtained from both parents - if both have authority, or guardian)*	yes<br>no<br><br>no
5.3.5 Incapacitated adults	

Participants are incapacitated/ decisionally incompetent adults *(if applicable, written informed consent will be obtained from legal representative)*	no

5.4 Recruitment and informed consent/objection

**5.4.1**	Retrospective study (tick all that apply)<br>☒ Not applicable (see section 5.2.2)<br>☐ Data will be copied from (electronic) patient records (e.g. 'EPD UMCG')<br>• <text on screening procedure, including informed consent procedure or give reasons if one or more participants will not be asked informed consent and underpin these with strong arguments><br>○ Total number of participants who will not be asked informed consent for screening:<br>○ Total number of UMCG participants who will not be asked informed consent for screening:<br>• <text on recruitment, including informed consent procedure or give reasons if one or more participants will not be asked informed consent and underpin these with strong arguments><br>○ Total number of participants who will not be asked informed consent:<br>○ Total number of UMCG participants who will not be asked informed consent:<br>☐ Data/biomaterials will be obtained from an already existing internal or external (UMCG/non-UMCG) bio- or databank (see Section 1. Study organization).<br>☐ Data/biomaterials will be obtained from a previous study ('FAIR data' - internal/external; see Section 1. Study organization).
**5.4.2**	Prospective study<br>☐ Not applicable (see section 5.2.2)<br>A call for participation is issued among the panel of the Dutch Patient Federation (23.000 members). Patients of the patient panel can read information about the study. Panel members can indicate if they want to participate in this study. When they are interested to participate they are asked to answer online some general question that cover the inclusion criteria. If they meet the inclusion criteria they will receive a link to the informed consent and the questionnaire. **(See Appendix III Informed consent and questionnaire)**

5.4.3 Objection (Registry)	
in case one or more participants will not be asked informed consent, the objection registry will be checked for these participants and the data from those who objected will be excluded from the analyses.	NA
5.4.4 Informed consent (IC): access to identifiable participant data	
in case one or more study team members will have access to direct/indirect identifiable participant data, informed consent will be/has been obtained for this access.	NA
5.4.5 IC: Collaboration with commercial parties	
In case of collaboration with commercial/profit organizations, informed consent will be/has been obtained for this type of collaboration	NA
5.4.6 IC: Linking with other registries	
In case the data will be linked with other registries, informed consent will be/has been obtained for this linkage(s)	NA
5.4.7 IC: Incidental findings	
In case there is a risk of incidental findings, informed consent will be/has been obtained to return findings to the participant	NA
5.4.8 IC: FAIR Data	

In case data collected for the present study will be shared for future studies, informed consent will be obtained for this	NA
<u>5.4.9 IC: other aspects</u>	
NA or <other relevant aspects of the study for which informed consent will be/has been obtained>	
<u>5.4.10 Withdrawal</u>	
• Can participants withdraw informed consent before publication and will all data/ biomaterials of that participant be destroyed<br>• Does the participant information letter contain information on how to withdraw	yes<br><br>yes

5.5 Research Data Management Plan (RDMP)

In this study the data will be collected, processed, and archived in accordance with the General Data Protection Regulation (GDPR) and the FAIR (Findable, Accessible, Interoperable, Reusable) principles under the responsibility of the Principal Investigator. A research data management plan (RDMP) <has been/~~will be~~> drawn up to describe the further operational details and procedures. <For details on the RDMP please find the RDMP enclosed in the Appendix>		
☐ the RDMP section below is completed<br>☒ a separate RDMP document will be attached to this protocol – **See appendix I**		
<u>5.5.1 Data collection</u>		
• Only essential baseline characteristics and data required to answer the research question(s) will be collected.		yes
• Tooling (eg. software and procedures) used for collecting, processing, analysing, and storing data will be compliant with the UMCG policy and Standard Operating Procedures in the UMCG Research Toolbox.		NA
Not a UMCG research		
<u>5.5.2 Anonymization and pseudonymization</u>		
• Data will be anonymised during data collection (i.e. data cannot be linked back to the participant)	Yes<br>**skip section 5.5.2**	~~No~~
<if no, explain why it is not possible to anonymise the data during collection>		
• Data will be pseudonymized by use of <a code list/an encryption key> during data collection.		NA
• Indirect and direct identifiable information collected will be minimized and only collected for the purpose of this study		NA
• Direct identifiable information (e.g. contact details, code list/encryption key/subject identification log) will be stored separately from pseudonymized data <in the electronic patient files (EPD), in an electronic file, on paper in the study file, other>		NA
<u>5.5.3 Data access (during the study)</u>		
• Direct identifiable information can only be accessed by the Principal Investigator and study delegates after authorization by the Principal Investigator.		yes
• Pseudonymized/anonymized data can only be accessed by the Principal Investigator and study delegates after authorization by the Principal Investigator.		yes

• Data roles, responsibilities, access and authorization - during the study and after study completion - will be managed and documented (e.g. in the RDMP, on study delegation log).	yes
5.5.4 Data sharing (during and after study completion)	
In case data (and biomaterials) will leave or enter the UMCG, will you contact the loket Contract Research to arrange the proper contracts?	no
External research	
5.5.5 Data storage (during and after study completion)	
• Digital data will be archived on the UMCG network complying with strict UMCG security and back-up policy.	no
External research and storaged at the Hanze University of Applied Sciences with strict Hanze University security and back-up policy.	
• Paper source data and study files will be archived within the UMCG facilities.	NA
• Source data, study files and digital data will be stored 15 years after the study is completed.	yes
5.5.6 Data re-use and access after completion of the present study ('FAIR data')	NA ☒ **skip section 5.5.6**
• Data will become available and shared for re-use and participants will be asked informed consent for this ('FAIR data')	NA
• Data will be made findable by including the description of the study (and type of data (i.e. metadata) in the UMCG FAIR data catalogue and other discipline specific catalogue(s).	NA
• Review procedure, conditions and agreements for re-use of data and access to data by other researchers will be drawn up.	NA
• For this study a discipline specific metadata standard will be chosen (i.e. to increase interoperability and re-use).	NA

5.6 Management of biomaterials

Will biomaterials be collected, processed, analyzed and/or stored for the purpose of this study	~~Yes~~	No **skip section 5.6**
5.6.1 Retrospective study (see sections 1, 5.2.2, and 5.4.1)<br>If biomaterials will be used from a secondary/further use biobank that has not been approved by the Board of Directors of the UMCG, how will be prohibited that biomaterials necessary for future diagnostic/treatment purposes will be used in the present study.		NA ☒
5.6.2 Biomaterials collection		
• Only biomaterials required to answer the research question(s) will be collected		NA
• What biomaterials will be collected<br><text>		
5.6.3 Pseudonymization and access to biomaterials		
• Does the storage unit of the biomaterials comprise information that the participant (in)directly identifies, other than the participant's number and / or the sample number.		NA
• Biomaterials can only be accessed by the Principal Investigator and study delegates after authorization by the Principal Investigator		NA
5.6.4 Sharing of biomaterials (during and after study completion)		
In case biomaterials (and data) will leave the UMCG, will you contact the loket Contract Research to arrange the proper contracts?		NA
5.6.5 Biomaterials storage (during and after study completion)		
• Where and how will the biomaterials be stored		
• Biomaterials will be stored 15 years after the study is completed		NA
• What will be done with the remaining biomaterials after study completion (eg. destroyed, returned to biobank/previous study, stored)		
5.6.6 Biomaterials re-use and access after completion of the present study		NA ☒ **skip section 5.6.6**
• Biomaterials will become available and shared for re-use and participants will be asked informed consent for this ('FAIR data')		NA
• Biomaterials will be made findable by including the description of the study (and type of biomaterials in the UMCG FAIR data catalogue and other discipline specific catalogue(s).		NA
• Review procedure, conditions and agreements for re-use of biomaterials and access to biomaterials by other researchers will be drawn up.		NA

5.7 Burden, Risks & Benefits (Prospective studies only)

• If participants are patients: Can be deviated from the standard care / diagnostic procedures (e.g. can medical treatment be postponed or limited)	NA
• Burden<br>Filling in the questionnaire takes approximately 20 minutes time of participants	

	Yes, minimal risk/burden	Yes, more than minimal risk/burden	No
• Will the participants risk any injuries and/or other discomfort when they participate in the proposed study	☐	☐	☒
• Participant benefits/reward/incentives:			

5.8 Incidental findings

	yes, minimal risk	yes, ≥ substantial risk	No
• Is there a risk of incidental findings?	☐	☐	☒
If yes,<br>○ Procedure to assess if a finding should be returned to the participant, or not<br>○ Procedure to inform the participant			

5.9 Data analysis

- Justification of sample size (e.g. power analysis)
  With an effect size of d = 0.4 this study requires a minimum of 834 participants to have a mean effect that has practical relevance (d = 0.4) with a power of 0.90 and an alpha of 0.05 (Brysbaert, 2019). This makes the probability 61% to establish the expected difference.
- Statistical analysis: Describe in detail the statistical analysis used to test the research question. See document *80D1.01 TPL Statistical Analysis Plan*, on DocPortal for guides lines.
  See appendix II

5.10 Participant information after the study

Will participants be informed about the study results	~~yes~~/no

5.11 Research revenue

In case the study will result in revenues (e.g. as a result of the use of data/biomaterials or successful licensing of intellectual property or manufactured products), will you contact the loket Contract Research to arrange the proper contracts?	NA/~~yes/no~~
Describe what will be done with the revenues.	

6. REFERENCES

Andrade, C. C., & Devlin, A. S. (2015). Stress reduction in the hospital room: Applying Ulrich's theory of supportive design. *Journal of Environmental Psychology*, *41*, 125–134.

Andrade, C. C., Devlin, A. S., Pereira, C. R., & Lima, M. L. (2017). Do the hospital rooms make a difference for patients' stress? A multilevel analysis of the role of perceived control, positive distraction, and social support. *Journal of Environmental Psychology*, *53*, 63–71.

Becker, F., & Parsons, K. S. (2007). Hospital facilities and the role of evidence-based design. *Journal of Facilities Management*, *5*(4), 263–274. https://doi.org/10.1108/14725960710822259

Dijkstra, K. (2009). *Understanding healing environments: Effects of physical environmental stimuli on patients' health and well-being*. https://doi.org/10.1177/193758671400800116

Larsen, L. S., Larsen, B. H., & Birkelund, R. (2014). A companionship between strangers-the hospital environment as a challenge in patient-patient interaction in oncology wards. *Journal of Advanced Nursing*, *70*(2), 395–404. https://doi.org/10.1111/jan.12204

Marteau, T. M., & Bekker, H. (1992). The development of a six-item short-form of the state scale of the Spielberger state-trait anxiety inventory (STAI). *Britisch Journal of Clinical Psychology*, *31*, 301–306.

Pluess. (2013). Sensory-Processing Sensitivity: A potential mechanism of differential susceptibility. *Presented at the Society for Child Development*.

Suess, C., & Mody, M. (2018). The influence of hospitable design and service on patient responses. *Service Industries Journal*, *38*(1–2), 127–147. https://doi.org/10.1080/02642069.2017.1385773

Taylor, E., Card, A. J., & Piatkowski, M. (2018). Single-occupancy patient rooms: A systematic review of the literature since 2006. *Health Environments Research & Design Journal*, *11*(1), 85–100. https://doi.org/10.1177/1937586718755110

Thompson, E. R. (2007). Development and validation of an internationally reliable short-form of the positive and negative affect schedule (PANAS). *Journal of Cross-Cultural Psychology*.

Ulrich, R. (1991). Effects of interior design on wellness: Theory and recent scientific research. *Journal of Healthcare Interior Design*, 97–109.

Ulrich, R., Quan, X., Zimring, C., Joseph, A., & Choudhary, R. (2004). *The role of the physical environment in the hospital of the 21st century: A once-in-a-lifetime opportunity*.

Watson, D., & Clark, L. A. (1994). *The PANAS-X: Manual for the positive and negative affect schedule - expanded form*.

Zijlstra, E. (2021). *The impact of the hospital environment: Understanding the experience of the patient journey*. https://www.hanze.nl/assets/kc-noorderruimte/facility-management/Documents/Public/Thesis Emma Zijlstra - The impact of the hospital environment %28excl. ch 6%29.pdf

7. APPENDICES

Appendix I: Informed Consent and Questionnaire

Appendix II: Data Management Plan

Appendix III: Statistical Analysis Plan

Appendix I Informed Consent and Questionnaire

Toestemmingsverklaring

Voor deelname aan het wetenschappelijke onderzoek:

Beleving Ziekenhuiskamer

Geachte heer/mevrouw,

U wordt gevraagd deel te nemen aan een wetenschappelijke studie. Deelname is vrijwillig. Voor deelname is uw schriftelijke toestemming vereist.

Voordat u beslist of u aan dit onderzoek wilt deelnemen, krijgt u uitleg over wat het onderzoek inhoudt. Lees deze informatie zorgvuldig door en vraag de onderzoeker om uitleg als u vragen heeft ( ). U kunt het ook bespreken met uw partner, vrienden of familie.

Algemene informatie

De Hanzehogeschool Groningen en het Universitair Medisch Centrum Groningen doen onderzoek naar het ontwerp van ziekenhuiskamers in Nederland.

Doel van het onderzoek

Het doel van dit onderzoek is om de ziekenhuiskamers te verbeteren voor patiënten. Daarom zijn wij benieuwd naar uw ervaringen en mening over een ziekenhuiskamer.

Wat betekent deelname:

Voor dit onderzoek zijn wij op zoek naar deelnemers die een vragenlijst willen invullen die ongeveer 20 minuten duurt. Bent u de afgelopen 5 jaar opgenomen geweest in een ziekenhuis in Nederland? Bent u opgenomen geweest voor minimaal 1 nacht? Dan kunt u deelnemen aan de vragenlijst over ziekenhuiskamers. Het maakt niet uit of u in een 1-persoonskamer of meerpersoonskamer opgenomen bent geweest.

In deze vragenlijst krijgt u foto's en een filmpje te zien van een ziekenhuiskamer. Er worden vragen gesteld over uzelf en over uw ervaringen en mening over een ziekenhuiskamer.

Als u niet wilt deelnemen of wilt stoppen met uw deelname aan het onderzoek

Het is aan u om te beslissen of u al dan niet aan het onderzoek deelneemt. Deelname is vrijwillig. Als u aan het onderzoek deelneemt, kunt u altijd van gedachten veranderen en besluiten te stoppen, op elk moment tijdens de vragenlijst. U kunt dan de vragenlijst afsluiten. De gegevens die tot dat moment zijn verzameld, zullen nog steeds voor het onderzoek worden gebruikt.

Einde van de vragenlijst

Uw deelname aan de studie stopt wanneer u de vragenlijst heeft beëindigd of zelf heeft besloten te stoppen.

Gebruik en opslag van uw gegevens

Voor dit onderzoek worden uw persoonsgegevens gebruikt en bewaard. Het gaat om gegevens zoals uw naam en e-mailadres. Het verzamelen, gebruiken en bewaren van deze gegevens is nodig om de vragen die in dit onderzoek worden gesteld te kunnen beantwoorden en de resultaten te kunnen publiceren. Wij vragen voor het gebruik van uw gegevens uw toestemming.

Om uw privacy te beschermen krijgen uw gegevens een code. Uw naam en andere gegevens die u direct kunnen identificeren worden daarbij weggelaten. In rapporten en publicaties over het onderzoek zijn de gegevens niet tot u te herleiden.

Intrekken toestemming

U kunt u toestemming voor het gebruik van de vragenlijstgegevens op elk moment intrekken. De onderzoeksgegevens die zijn verzameld tot het moment waarop u uw toestemming intrekt, worden nog wel voor het onderzoek gebruikt.

Heeft u vragen?
Bij vragen over het onderzoek kunt u contact opnemen met Sjoukje van Dellen (
).

Klik op 'volgende' om verder te gaan in het formulier.

Wilt u deelnemen aan de vragenlijst over ziekenhuiskamers?	Ja<br>Nee

Hartelijk dank

Hartelijk dank dat u wilt deelnemen aan de vragenlijst over ziekenhuiskamers.

Als u aan het eind van dit formulier uw gegevens invult en op de knop 'Voltooien' klikt, dan sturen we u binnenkort de vragenlijst toe.

Klik op 'volgende' om verder te gaan in het formulier.

Vragenlijst

De Hanzehogeschool Groningen en het Universitair Medisch Centrum Groningen doen onderzoek naar het ontwerp van nieuwe ziekenhuiskamers. Het doel van dit onderzoek is om de ziekenhuiskamers te verbeteren voor patiënten. Daarom zijn wij benieuwd naar uw ervaringen en mening over een patiëntenkamer in het ziekenhuis.

Bent u de afgelopen 5 jaar opgenomen geweest in een ziekenhuis in Nederland? Bent u opgenomen geweest voor minimaal 1 nacht? Dan kunt u deelnemen aan de vragenlijst over ziekenhuiskamers. Het maakt niet uit of u in een 1-persoonskamer of meerpersoonskamer opgenomen bent geweest.

Hoe lang is het geleden dat u opgenomen bent geweest in het ziekenhuis voor minimaal 1 nacht?	In de afgelopen 2 jaar<br>3 tot 5 jaar geleden<br>Langer dan 5 jaar geleden
Bent u opgenomen geweest op een psychiatrische afdeling?	Ja (niet de doelgroep)<br>Nee
Bent u opgenomen geweest in een revalidatiekliniek?	Ja (niet de doelgroep)<br>Nee
Bent u opgenomen geweest voor een geboorte van een kind?	Ja (niet de doelgroep)<br>Nee

Je valt helaas buiten de doelgroep van dit onderzoek als

- u op een psychiatrische afdeling opgenomen bent geweest
- u in een revalidatiekliniek opgenomen bent geweest
- u opgenomen bent geweest i.v.m. een geboorte
- het langer dan 5 jaar geleden is dat u opgenomen bent geweest in het ziekenhuis

Hartelijk dank voor uw interesse.

Klik op ‘volgende’ om verder te gaan in het formulier.

Wilt u deelnemen aan de vragenlijst over ziekenhuiskamers?	Ja<br>Nee

Hartelijk dank

Hartelijk dank dat u wilt deelnemen aan de vragenlijst over ziekenhuiskamers.

Als u aan het eind van dit formulier uw gegevens invult en op de knop ‘Voltooien’ klikt, dan sturen we je binnenkort de vragenlijst toe.

Start vragenlijst

Algemene vragen

1. Met welk geslacht identificeert u zichzelf?	Man<br>Vrouw<br>Beide<br>Geen van beide
2. Wat is uw leeftijd?	18 -29 jaar<br>30 – 44 jaar<br>45 – 59 jaar<br>60 – 75 jaar<br>Ouder dan 75
3. In welke provincie woont u?	Friesland<br>Groningen<br>Drenthe<br>Flevoland<br>Overijssel<br>Utrecht<br>Gelderland<br>Noord-Holland<br>Zuid-Holland<br>Zeeland<br>Noord-Brabant<br>Limburg
4. Wat is uw hoogst genoten opleiding?	Basisschool<br>LBO, MAVO, VMBO, HAVO, VWO, MBO<br>HBO, WO, PhD
5. Wat is uw samenstelling van uw huishouden?	Met partner<br>Met kinderen<br>Met partner en kinderen<br>Zonder partner en kinderen
6. Wat is uw werksituatie?	Fulltime of parttime werkzaam<br>Geen werk (huisvrouw/-man)<br>Werkzoekend<br>Gepensioneerd<br>Ziek<br>Anders
7. Waar bent u geboren?	Nederland<br>West-Europa<br>Oost-Europa<br>Midden-Oosten<br>Azië<br>Afrika<br>Noord-Amerika<br>Midden-Amerika<br>Zuid-Amerika<br>Canada<br>Australië / Nieuw Zeeland

Eerdere ziekenhuiservaring

Onderstaande vragen gaan over uw ervaringen tijdens u laatste opname in het ziekenhuis.

8. Wanneer bent u voor het laatst opgenomen geweest in het ziekenhuis?	In de afgelopen 2 jaar<br>3 tot 5 jaar geleden<br>Langer dan 5 jaar geleden
9. Hoeveel nachten verbleef u in het ziekenhuis tijdens deze opname?	…. nachten
10. Wat was de urgentie van uw opname?	Spoed<br>Geen spoed
11. Voor wat voor diagnose bent u opgenomen geweest?	Oncologie (behandelen van kanker)<br>Hart- en vaatziekten<br>Longen en luchtwegen<br>Maag-, darm- en leverziekten<br>Blaas, urinewegen, nieren (urologie)<br>Gynaecologie<br>Gewrichten, botten, spieren en pezen (orthopedie)<br>Hersenen, ruggenmerg, zenuwen en/of spieren (neurologie)<br>KNO (keel, neus en oorheelkunde)<br>Gebit, kaak en aangezicht<br>Ogen<br>Ouderdom<br>Overgewicht<br>Anders, namelijk….
12. Heeft u tijdens deze opname een operatieve behandeling ondergaan?	Ja (vraag 13)<br>Nee (vraag 14)
13. Wat voor soort verdoving heeft u gekregen voor uw operatie?	Plaatselijke verdoving<br>Ruggenprik<br>Algehele narcose<br>Anders, namelijk…
14. Heeft u tijdens deze opname op de IC gelegen?	Ja<br>Nee
15. Heeft u complicaties gekregen tijdens deze opname?	Nee<br>Trombose<br>Luchtlekkage<br>Longontsteking<br>Nabloeding<br>Bloeduitstorting<br>Infectie<br>Heesheid<br>Anders, namelijk…..
16. Op wat voor ziekenhuiskamer heeft u gelegen? (meerdere antwoorden mogelijk)	1-persoonskamer<br>2-persoonskamer<br>3-persoonskamer<br>4-persoonskamer<br>Anders, namelijk….
17. Achteraf bekeken, wat voor ziekenhuiskamer had u voorkeur?	1-persoonskamer<br>2-persoonskamer

	3-persoonskamer<br>4-persoonskamer<br>Anders, namelijk….
18. Hoe herinnert u zich u (laatste) ziekenhuisopname in het algemeen?	1 (zeer negatief); 10 (zeer positief)
19. Hoeveel stress ervaarde u tijdens uw laatste ziekenhuisopname?	1 (zeer veel stress); 10 (helemaal geen stress)
20. Kunt u een korte omschrijving geven van de ziekenhuiskamer tijdens uw laatste ziekenhuisopname?	Open antwoord
21. Wilt u verder nog wat kwijt over uw (laatste) ervaringen tijdens uw ziekenhuisopname?	Open antwoord
22. Ziekenhuisopnames: Bent u vaker dan 1 keer opgenomen geweest in het ziekhuis? Zo ja, hoe vaak bent u in uw leven opgenomen geweest in het ziekenhuis?	…. keer

Gezondheid (SF-12 en SHMS)

Self-rated general health (SF-12) en Self-rated mental health - SHMS (Ahmad et al., 2014)

23. Hoe zou u over het algemeen uw gezondheid op dit moment noemen?	Uitstekend<br>Zeer goed<br>Goed<br>Matig<br>Slecht
24. Hoe zou u over het algemeen uw geestelijke gezondheid op dit moment noemen?	Uitstekend<br>Zeer goed<br>Goed<br>Matig<br>Slecht
25. Heeft u één of meerdere chronische aandoeningen?	Geen chronische aandoening<br>Eén chronische aandoening<br>Meer dan 1 chronische aandoening

Gevoelens en emoties

PANAS short scale (Thompson, 2007; Watson & Clark, 1994)

Hieronder ziet u een aantal woorden die verschillende gevoelens en emoties beschrijven. Wilt u ieder lezen en dan aangeven in welke mate u zich de afgelopen twee weken zo heeft gevoeld.

	Nauwelijks of helemaal niet	Een beetje	Gemiddeld	Nog al	In sterke mate
26. Vastberaden	1	2	3	4	5
27. Aandachtig	1	2	3	4	5
28. Alert	1	2	3	4	5

29. Geinspireerd	1	2	3	4	5
30. Actief	1	2	3	4	5
31. Bang	1	2	3	4	5
32. Nerveus	1	2	3	4	5
33. Van streek	1	2	3	4	5
34. Beschaamd	1	2	3	4	5
35. Vijandig	1	2	3	4	5

Sensitiviteit (HSC scale (Pluess, 2013)**)**

Beantwoord iedere vraag dat u gevoel u ingeeft.

	Hele maal niet			Matig			Heel erg
36. Ik ben me bewust van subtiele signalen in mijn omgeving	1	2	3	4	5	6	7
37. Ik raak gemakkelijk overvoerd door dingen als fel licht, sterke geuren, grove weefsels of harde sirenes	1	2	3	4	5	6	7
38. Ik heb een rijke en complexe innerlijke belevingswereld	1	2	3	4	5	6	7
39. Ik voel me opgejaagd als ik veel moet doen in korte tijd	1	2	3	4	5	6	7
40. Ik kan diep geroerd raken door kunst of muziek	1	2	3	4	5	6	7
41. Ik raak geïrriteerd als mensen proberen me te veel dingen tegelijk te laten doen	1	2	3	4	5	6	7
42. Ik kijk uit principe niet naar gewelddadige films of tv-shows	1	2	3	4	5	6	7
43. Ik voel me ongemakkelijk als er veel om me heen gebeurt	1	2	3	4	5	6	7
44. Veranderingen in mijn leven brengen me van mijn stuk	1	2	3	4	5	6	7
45. Ik heb een neus voor delicate geuren, smaken, geluiden en kunstwerken en geniet daarvan	1	2	3	4	5	6	7
46. Ik voel mij niet op mijn gemak bij harde geluiden	1	2	3	4	5	6	7
47. Als ik moet concurreren of op mijn vingers word gekeken, word ik zo nerveus of gespannen dat mijn prestaties veel minder zijn dan gewoonlijk	1	2	3	4	5	6	7

[START EXPERIMENT]

- Weergave film van ziekenhuiskamer (ca. 2 minuten)
- Foto's laten zien van ziekenhuiskamer

Gemoedstoestand: (laat een foto zien)

STAI-scale (Marteau & Bekker, 1992)

Stelt u zich voor dat u tijdens uw ziekenhuisopname in deze kamer zou komen te liggen voor uw herstel:

	1<br>Geheel niet	2<br>Een beetje	3<br>Tamelijk veel	4<br>Zeer veel
48. In welke mate zou u zich dan kalm voelen in deze kamer?	1	2	3	4
49. In welke mate zou u zich dan gespannen voelen in deze kamer?	1	2	3	4
50. In welke mate zou u in de war zijn in deze kamer in deze kamer?	1	2	3	4
51. In welke mate zou u dan ontspannen zijn in deze kamer?	1	2	3	4
52. In welke mate zou u zich dan tevreden voelen in deze kamer?	1	2	3	4
53. In welke mate zou u zich dan zorgen maken in deze kamer?	1	2	3	4

54. Hoeveel stress verwacht u te hebben als u in deze kamer zou liggen tijdens een opname op een verpleegafdeling?	1: Zeer veel stress, 10: Helemaal geen stress

Over de kamer: (laat een foto zien)

SHEDS – Supportive Hospital Environment Design Scale (positive distraction, sense of control, social support)

	Zeer mee oneens	Een beetje mee oneens	Niet mee oneens/ eens	Een beetje mee eens	Zeer mee eens
55. In deze ziekenhuiskamer ben ik in staat de omgeving te controleren.	1	2	3	4	5
56. Ik heb controle over de fysieke kenmerken en materialen van mijn ziekenhuiskamer	1	2	3	4	5

57. Ik kan keuzes maken over de fysieke kenmerken van mijn ziekenhuiskamer.	1	2	3	4	5
58. In deze ziekenhuiskamer kan ik dingen naar mijn behoefte aanpassen, opnieuw indelen en reorganiseren als het nodig is.	1	2	3	4	5
59. Ik bepaal de organisatie/uitstraling van mijn ziekenhuiskamer.	1	2	3	4	5
60. In deze ziekenhuiskamer kan ik praten/"samenkomen" met familie en vrienden die mij bezoeken.	1	2	3	4	5
61. Mijn familie en vrienden zouden zich op hun gemak voelen in deze ziekenhuiskamer.	1	2	3	4	5
62. In deze ziekenhuiskamer zou ik kunnen genieten van het gezelschap van bezoekende familie en vrienden.	1	2	3	4	5
63. Deze ziekenhuiskamer biedt een ondersteunende omgeving voor bezoek van familie en vrienden.	1	2	3	4	5
64. In deze kamer wordt mijn aandacht naar interessante dingen getrokken.	1	2	3	4	5
65. In deze ziekenhuiskamer zijn er voorwerpen die mijn aandacht trekken.	1	2	3	4	5
66. In deze ziekenhuiskamer word ik door de omgeving opgezogen.	1	2	3	4	5
67. Er is in deze ziekenhuiskamer genoeg waar ik naar wil blijven kijken.	1	2	3	4	5

Algemeen:

[laat een foto zien van de kamer bij de vraag]

68. Hoe aangenaam vindt u de ziekenhuiskamer eruit zien?	1: niet aangenaam, 10: zeer aangenaam
69. Hoe aangenaam vindt u de badkamer eruit zien?	1: niet aangenaam, 10: zeer aangenaam

Inrichting van de kamer:

Atmospherics of the healthcare environment (Suess & Mody, 2018)

	Volledig oneens						Volledig eens
70. De sfeerverlichting zorgt voor een aangename sfeer	1	2	3	4	5	6	7
71. De kleuren van de muren, vloeren en plafonds zijn aangenaam	1	2	3	4	5	6	7
72. De algemene decoratie is aantrekkelijk	1	2	3	4	5	6	7
73. Er zijn voldoende planten en bloemen	1	2	3	4	5	6	7
74. De schilderijen en foto's zijn aantrekkelijk	1	2	3	4	5	6	7
75. Er is genoeg kunst en decoratie	1	2	3	4	5	6	7
76. De inrichting is comfortabel	1	2	3	4	5	6	7
77. De inrichting is visueel aantrekkelijk	1	2	3	4	5	6	7

Hartelijk dank!

Appendix II Data Management Plan

The impact of the hospital inpatient room on patients' stress

A Data Management Plan created using DMPonline

Creator: Emma Zijlstra

Affiliation: Hanze University of Applied Sciences

Funder: Hanze University of Applied Sciences

Template: Data management plan of the Hanze University of Applied Sciences Groningen

Project abstract:
Background: Many patients experience stress during hospitalization (Andrade & Devlin, 2015; Zijlstra, 2021). The theory of supportive design conceptualizes that the physical environment improves the sense of control, access to social support and positive distraction which positively effects patients' well-being (Ulrich, 1991).
Main research question:What is the effect of a single-bed inpatient room design on patients' stress?
Design (including population, confounders/outcomes): This study is an online randomized controlled trial with a between-subjects design. Participants are assigned to one of four conditions, namely the current patient room, a patient room with architectural changes, a patient room with interior changes, a patient room with architectural and interior changes. Participants are included when they have been hospitalized in the last 2 years in a Dutch hospital after surgery (excluding oncology surgery). Perceived stress, sense of control, social support, positive distraction and pleasantness of the room are measured using a questionnaire.
Expected results: It is expected that participants exposed to a new design features anticipated less stress than participants that are exposed to the current patient room. It is expected that this positive effect is mediated by sense of control, positive distraction, social support, and perceived pleasantness. It is also expected that participants who score higher in environmental sensitivity perceive more sense of control, positive distraction, social support and perceived pleasantness in the new design and, therefore, perceive less stress.

ID: 98838

Start date: 16-05-2022

End date: 30-06-2023

Last modified: 04-05-2022

The impact of the hospital inpatient room on patients' stress

1. General Information

Name of the researcher(s):

Dr. Emma Zijlstra
Dr. Mark Mobach
Drs. Sjoukje van Dellen

Name of the group/project:

Hospital Patient Room of the Future

Describe the research, including purpose and the (possible) impact.

Background

Many patients experience stress during hospitalization (Andrade & Devlin, 2015; Zijlstra, 2021). The theory of supportive design conceptualizes that the physical environment improves the sense of control, access to social support and positive distraction which positively effects patients' well-being (Ulrich, 1991).

- **Main research question**

What is the effect of a single-bed inpatient room design on patients' stress?

- **Design (including population, confounders/outcomes)**

The study is an online randomized controlled trial with a between-subjects design. Participants are assigned to one of four conditions, namely the current patient room, a patient room with architectural changes, a patient room with interior changes, a patient room with architectural and interior changes. Participants are included when they have been hospitalized in the last 2 years in a Dutch hospital after surgery (excluding oncology surgery). Perceived stress, sense of control, social support, positive distraction and pleasantness of the room are measured using a questionnaire.

- **Expected results**

It is expected that participants exposed to a new design features anticipated less stress than participants that are exposed to the current patient room. It is expected that this positive effect is mediated by sense of control, positive distraction, social support, and perceived pleasantness. It is also expected that participants who score higher in environmental sensitivity perceive more sense of control, positive distraction, social support and perceived pleasantness in the new design and, therefore, perceive less stress.

- **Impact**

This study contributes to scientific knowledge with regard to the inpatient room design and the influence on patients' experiences and well-being. The findings of this study allows facility managers and designers to better understand the patients' experiences and to make better informed decisions about inpatient room designs.

Funder/Funding bodies:

- Not applicable

Partner organizations of the research project (include personal contact details):

University Medical Center of Groningen, Jan Bouwhuis, Director Facilities and Estates

Project duration:

15 may 2022 - 30 juni 2023

Date of this data management plan:

4 May 2022

Update (date) and version number of this data management plan:

20220504 V0.2

Names and roles (e.g. data steward) of the persons and departments you consulted on this plan:

- Data steward Sabine van der Ham (Research centre NoorderRuimte)

Date of consultation of this plan with the data steward:

22 April 2022

2. Preparation and planning

As a researcher you have the responsibility to conduct the research in an integer, honest, independent and transparant way.
Take into account the following laws and regulations. Is the research project tied to/subject to any of these laws or regulations?

- General Data Protection Regulation
- Netherlands Code of Conduct for Research Integrity

Which (additional) resources do you need to manage the research data?

- Software

Which (new) technical tools and/or skills are required for this research project?

- Specific tool and skills for collecting quantitative research data, please specify
- Quantitative analysis skills and/or specific tool, app, software, namely

Qualtrics is used for collecting quantitative research data
SPSS is used for analyzing the quantitative data
Atlas.ti is used for analysing the qualitative data

Who owns/controls the data?

Hanze University of Applied Sciences owns/controls the research data.

How are the files and folders of the research project organized?

000-Research preparation
100-Finances
200-Correspondence
300-Research implementation
400-Research data
500-Research management
600-Reports
700-Publications, presentations, media
800-Work documents
900-Overview archive locations

Which communication tool/platform is used for collaborating with co-workers/researchers and/or third parties?

All anonymized data will be processed and used by the researcher at Hanzehogeschool Groningen. This data is stored on a secure network on the Research Drive. The Research Drive is offered through SURF and compliant with the required laws and regulations, such as the AVG/GDPR.

3. Data collection, data processing and data organization

How will the data be collected or produced?

A call for participation is issued among the panel of the Dutch Patient Federation (23.000 members). Panel members can indicate if they want to participate in this study. Patients of the patient panel can read information about the study. When they are interested to participate they are asked to answer online some general question that cover the inclusion criteria. If they meet the inclusion criteria they will receive a link to the questionnaire. The data of the questionnaire will be collected by using Qualtrics (RUG/UMCG).

Who is responsible for which part of data management, during the research project?

Data Management	Researcher/Co-researchers (Names)	Responsible Supervisor (Name)
Who is collecting the data?	Emma Zijlstra	Mark Mobach
Who is processing and/or analysing the data?	Emma Zijlstra & Sjoukje van Dellen	Mark Mobach
Who is **managing** the research data (e.g. storage, sharing, and archiving of the data and informing all research partners)	Emma Zijlstra & Sjoukje van Dellen	Mark Mobach
Who is **erasing** the data?	Emma Zijlstra	Mark Mobach

Is existing data (from other research) being reused?

- No

Is (sensitive) personal data collected?

- No

Provide an overview of the expected types of research data (text, audio, video etc), software choices, data size and growth, the storage location and the back-up planning.

Phase	Specification of type of research data (text, audio, video etc.)	software choice(s) / platforms	file extension (.pdf, .jpeg .png, .csv, .mxf, .x3d, .svg, .dxf, etc)	encryption	storage location(also country) and back-up
Raw data	survey	Qualtrics	.csv		Onedrive and Research drive, European storage location
Processed data	survey	SPSS	.sav	X	Onedrive and Research drive, European storage location
Models/code (e.g. data from simulations)	survey	SPSS	.sav	X	Onedrive and Research drive, European storage location

Do you only collect data that contributes to answering the research question?

- Yes

A questionnaire is used to measure the following variables:
Primary outcomes: Patients' anxiety and patients' stress
Mediators: Sense of control, social support, positive distraction, atmospherics of the healthcare environment, and pleasantness of the room
Moderator: Environmental sensitivity
Participants characteristics: Demographics, hospitalization history, self-reported psychophysical well-being

Are trade secrets or undisclosed know-how related to or subject of the research?

- No

If the research results in innovative use or application of new technological solutions, Intellectual Property Rights (IPR) have to be acknowledged. Does the research result in innovative use or application of new technological solutions?

- No

How do you name the data files in a consistent and logical way during the research?

The following elements are included in the file names:
date(YYYYMMDD), consistent title, initials editors, version number (v01)
The following structure is used for data files:
00041_set up_data_collection
00042_data management planning
00043_collected raw data
00044_enriched data sets
00045_analysed data sets
00046_processed data sets
00047_datasets versions
00048_published data sets
00049_external sharing

How are the versions of the files, folders, documents and records controlled?

- all concept versions until the definitive version receive the begin digit 0 (zero);
- all modified versions receive a period after the begin digit, followed by an ascending number. The first concept will get the sequential number 0.1, after a modification the number 0.2, et cetera;
- all versions from the definitive version onwards receive a begin digit 1 (one), the first definitive version 1.0. All versions with small modifications receive a new ascending number after the period. The first modified version will get the sequential number 1.1, after a modification the number 1.2, et cetera;
- versions in which considerable modifications are made (for example adding or deleting a chapter) will receive a new sequential begin digit, starting with 2.0, et cetera.

4. Data preservation, data dissemination (FAIR data) and data publishing

What data will be made available for re-use in a data repository?

- It is not possible to make any research data of the project available (please specify)

In which data repository is it possible to find the final data of the research project for further research?

NA

Are the metadata (data about research data) of the research findable in a searchable online resource e.g. a catalogue or a data repository?

NA

Which metadata standard is used to make the final data findable?

NA

A Persistent Identifier (PI) has to be assigned to the (meta)data to be findable to humans and computers. In most cases the selected data repository will generate a Persistent Identifier, e.g. a Digital Object Identifier (DOI).
Is a persistent identifier assigned to the (meta)data?

NA

Are the research data accessible for researchers (humans) and computers (machines)?

- No, the research data aren't accessible, because...

The data is not shared with outsiders, because this is not required.

Can the final data be retrieved by a recognized standard and/or protocol?

NA

When will the data be accessible?

NA

Does the access procedure include authentication and authorization steps, if necessary?

NA

Do the final data have a clear and explicit data usage license to make them as reusable as possible?

NA

Are the data filed in recognized formats to make them interoperable?
Please add the required information in the table below.

Final data (multiple datasets)	Specification of type of research data per set	software choice(s)/platforms per set	file extension (.pdf/a .html, MATLAB, .csv, .sql, .por, .jpeg, .png, .mkv, .mxf, .x3d, .svg, .dxf, etc)	file size
Dataset	Survey data	Excel	.xls	unknown

Are controlled vocabularies, keywords, thesauri or ontologies used to make the final data interoperable?

NA

Are qualified references and links to other related data provided?

NA

The data description is an important step, when depositing the data in a data repository. Well-described data can be found and re-used in a better way.
With which relevant attributes are the data described? Elements for accurate and complete data description are mentioned in the Guidance

NA

Do the data and metadata meet relevant domain standards?

NA

Which data will be selected for long-term preservation?

- All data resulting from the research project will be preserved

All data will be preserved for 15 years on the Hanze Research Drive.

How and by whom have the data been prepared for depositing as final data in a data repository? It is also important to assign a person who can determine to grant reuse and who serves as a broker.

NA

What is the planning of the erasure of the research data in a data repository?

NA

Once the data is deposited in a data repository, are there restrictions to data sharing or embargo reasons?

NA

Where will the data be published?

NA

If applicable, describe what the strategy is for publishing the analysis software that will be generated in this project?

NA

Appendix III Statistical Analysis Plan

The Impact of the Hospital Inpatient Room on Patients' Stress

STATISTICAL ANALYSIS PLAN

Version: 1.0

Date of document: 02-05-2022

1. Introduction

The purpose of the statistical analysis plan (SAP) is to provide a comprehensive and detailed description of the rationale, methods and reporting of the data analysis for the Hospital Patient Room of the Future study.

2. Protocol summary

2.1 Study design

The study is an online randomized controlled trial with a between-subjects design with four levels. Participants are assigned to one of four conditions, namely the current patient room, a patient room with architectural changes, a patient room with interior changes, a patient room with architectural and interior changes. Participants are included when they have been hospitalized in the last 5 years in a Dutch hospital.

With an effect size of d = 0.4 this study requires a minimum of 834 participants to have a mean effect that has practical relevance (d = 0.4) with a power of 0.90 and an alpha of 0.05 (Brysbaert, 2019). This makes the probability 61% to establish the expected difference.

Eligiblity criteria of participants is shown in Table 1

Table 1 Eligibility criteria (inclusion and exclusion criteria)

**Inclusion criteria**	**Exclusion criteria**
18 years or older	Have been hospitalized at a psychiatric ward
Have been hospitalized for at least 1 night in the last 5 years in the Netherlands	Have been hospitalized on a IC unit
Sufficient knowledge of the Dutch language.	Have been hospitalized at a revalidation clinic
	Have been hospitalized with birth as reason

2.2 Study flow chart

Figure 1 Study flow chart

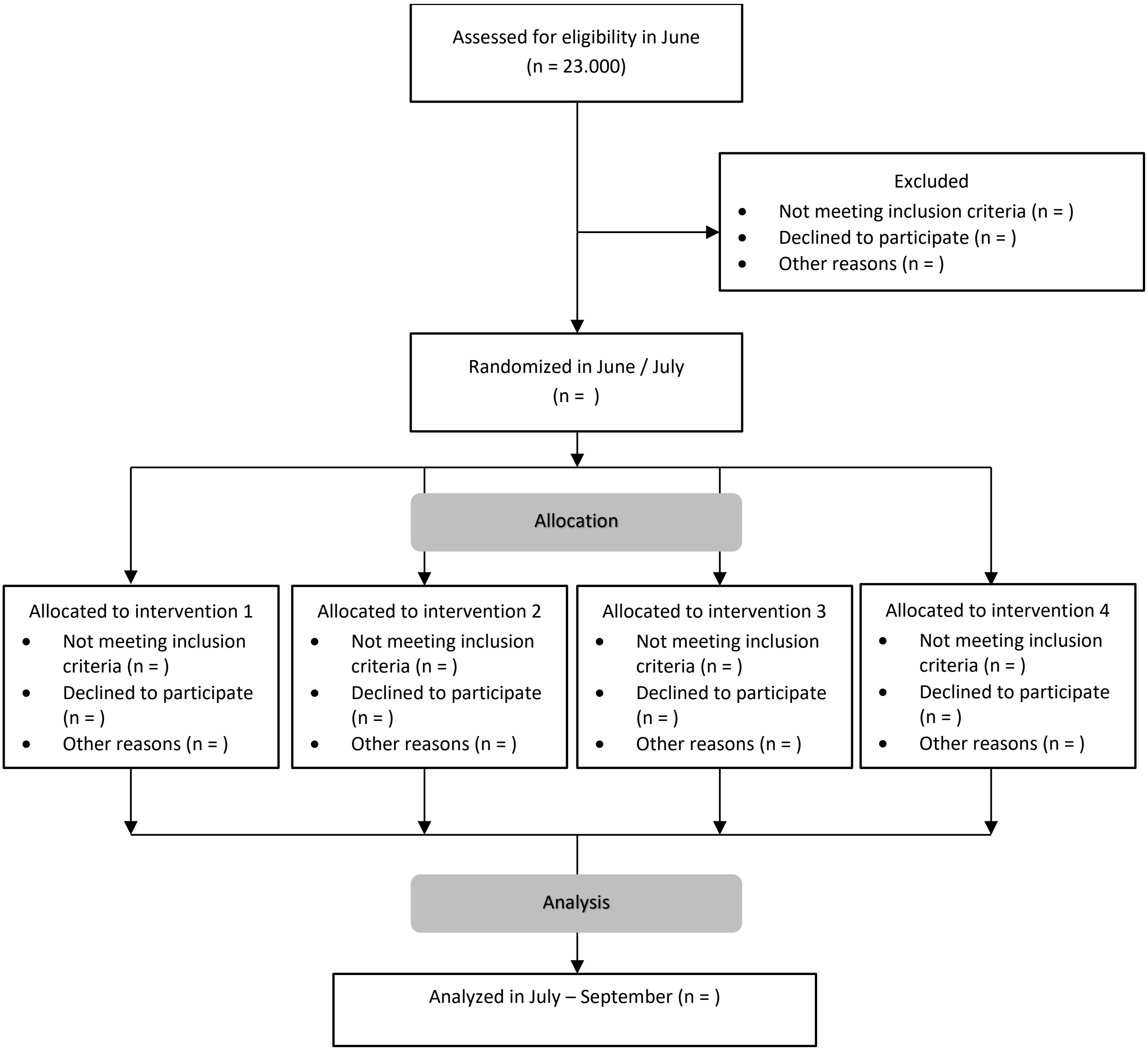

3. Study objective and measures

The primary objective of the study is to evaluate the effect of the design of a single bed hospital inpatient room on patients' stress.

3.1 Study outcome - general considerations

It is expected that participants exposed to new design features anticipated less stress than participants that are exposed to the current patient room. It also expected that participants exposed to more variation in design feature variations anticipated less stress than participants that are exposed to less variation in design feature variations. It is expected that this positive effect is mediated by sense of control, positive distraction, social support, and perceived pleasantness. It is also expected that participants who score higher in environmental sensitivity perceive more sense of control, positive distraction, social support and perceived pleasantness in the new design and, therefore, perceive less stress.

3.2 Primary outcome parameters

Patients' anxiety – Patients' anxiety is assessed by the short State-Trait Anxiety Inventory (Marteau and Bekker, 1992). This six-item short form (STAI-6) measures the level of stress at the specific moment while they have seen the intervention. Each item is measured from 1 (not at all) tot 4 (very much). The positive items will be reversed. A higher score reflects more patients' stress. Total score is 6 – 24.

Patients' stress – Patients' stress is assessed by a single item measurement. This item is measured on a 10-point bipolar scale ranging from (1) 'extremely' to (1) 'not at all'.

3.3 Secondary outcome parameters

Mediators

Sense of control – Sense of control is assessed by the Supportive Hospital Environment Design Scale (Andrade and Devlin, 2015; Andrade *et al.*, 2017). This 5-item scale (SHEDS) measures the expected perceived sense of control in the patient room. Each item is measured from (1) 'strongly disagree' to (5) 'strongly agree'. A higher score reflects more expected sense of control. Total score is 5 – 25.

Positive distraction – Positive distraction is assessed by the Supportive Hospital Environment Design Scale (Andrade and Devlin, 2015; Andrade *et al.*, 2017). This 4-item scale (SHEDS) measures the expected perceived positive distraction in the patient room. Each item is measured from (1) 'strongly disagree' to (5) 'strongly agree'. A higher score reflects more expected perceived positive distraction. Total score is 4 – 20.

Social support – Social support is assessed by the Supportive Hospital Environment Design Scale (Andrade and Devlin, 2015; Andrade *et al.*, 2017). This 4-item scale (SHEDS) measures the expected social support in the patient room. Each item is measured from (1) 'strongly disagree' to (5) 'strongly agree'. A higher score reflects more expected social support. Total score is 4 – 20.

Perceived pleasantness of the room – Participants were asked to rate 2 items. The first item measures the pleasantness of the room and the second item measures the perceived pleasantness of the bathroom. Both items are measured on a 10-point bipolar scale ranging from (1) 'not pleasant' to (10) 'very pleasant'.

Atmospherics of the room – The atmospherics of the room is assessed by the Atmospherics of the Healthcare Environmental Scale (Suess and Mody, 2018). This 13-item scale measures the appeal of the patient room. Because five items did not measure the visually appeal of the room, these items were deleted for this study. Each item is measured from (1) 'strongly disagree' to (7) 'strongly agree'. A higher score reflects a room with more appeal. Total score is 8 – 56.

Moderator

Environmental sensitivity – When studying the effect of the design of the patient room, it is important to take individual differences into account. Not all individuals may respond as strongly to differences in the patient room. Environmental sensitivity is assessed by the Sensory Processing Scale (Aron and Aron, 1997; Pluess, 2013). This 12-item short-scale measures the level of sensitivity of the participants. A higher score reflects a stronger reaction to external environments. It is expected that the effect of the intervention will be stronger when participants score higher in environmental sensitivity. Each item is measured from (1) 'not al all' to (7) 'extremely'. Total score is 7 – 84.

4. Statistical methods and reporting

4.1 Sample size

With an effect size of d = 0.4 this study requires a minimum of 834 participants to have a mean effect that has practical relevance (d = 0.4) with a power of 0.90 and an alpha of 0.05 (Brysbaert, 2019). This makes the probability 61% to establish the expected difference.

4.2 Level of significance

For all hypothesis testing performed, a p-value of less than 0.05 will indicate statistical significance.

4.3 Randomization, database lock and unblinding

For this study we used an online randomized controlled trial. Participants were randomly assigned to one of the four conditions. The study analysis will be performed on the final data that will become available after the database is formally locked.

4.4 Study participants characteristics

A description of the study population (using the primary study analysis set) will be given using the study subjects characteristics at baseline. Included demographical baseline characteristics are shown in Table X, and baseline characteristics of last hospitalization are shown in Table X, and baseline characteristics of self-reported psychophysical well-being in Table X.

Table 2 Baseline data - demographics

	**Intervention 1**	**Intervention 2**	**Intervention 3**	**Intervention 4**
Gender<br>• Male<br>• Female<br>• Both<br>• Neither	Frequency (%)	Frequency (%)	Frequency (%)	Frequency (%)

Age	Mean (SD)	Mean (SD)	Mean (SD)	Mean (SD)
Province of residence<br>• Friesland<br>• Groningen<br>• Drenthe<br>• Flevoland<br>• Overijssel<br>• Gelderland<br>• Utrecht<br>• Noord-Holland<br>• Zuid-Holland<br>• Zeeland<br>• Noord-Brabant<br>• Limburg	Frequency (%)	Frequency (%)	Frequency (%)	Frequency (%)
Education<br>• Low<br>• Middle<br>• High	Frequency (%)	Frequency (%)	Frequency (%)	Frequency (%)
Household<br>• With partner<br>• With children<br>• With partner and children<br>• Without partner and children	Frequency (%)	Frequency (%)	Frequency (%)	Frequency (%)
Work situation<br>• Full time or part time<br>• Not working (housewife/ -man)<br>• Looking for work<br>• Retired<br>• Sick<br>• Other	Frequency (%)	Frequency (%)	Frequency (%)	Frequency (%)
Ethnicity (birthplace)<br>• Netherlands<br>• Western Europe<br>• Eastern Europe<br>• Middle East<br>• Asia<br>• Africa	Frequency (%)	Frequency (%)	Frequency (%)	Frequency (%)

• North America<br>• Central America<br>• South America<br>• Canada<br>• Australia / New Zealand				

Table 3 Baseline data – hospitalization history

	**Intervention 1**	**Intervention 2**	**Intervention 3**	**Intervention 4**
Time ago last hospitalization<br>• In the past 2 years<br>• 3 to 5 years ago<br>• More than 5 years ago	Frequency (%)	Frequency (%)	Frequency (%)	Frequency (%)
Reason last hospitalization<br>• Oncology (treating cancer)<br>• Cardiovascular diseases<br>• Lungs and airways<br>• Gastrointestinal and liver diseases<br>• Bladder, urinary tract, kidneys (urology)<br>• Gynecology<br>• Joints, bones, muscles and tendons (orthopedics)<br>• Brain, spinal cord, nerves and/or muscles (neurology)<br>• ENT (throat, nose and ear surgery)<br>• Teeth, jaw and face<br>• Eyes<br>• Age<br>• Overweight<br>• Other, namely....	Frequency (%)	Frequency (%)	Frequency (%)	Frequency (%)
Urgency last hospitalization<br>• Emergency<br>• No emergency	Frequency (%)	Frequency (%)	Frequency (%)	Frequency (%)
Type of anesthesia last hospitalization	Frequency (%)	Frequency (%)	Frequency (%)	Frequency (%)

• No anesthesia<br>• Local anesthesia<br>• Spinal puncture<br>• General anesthesia<br>• Other				
Complications last hospitalization<br>• No complications<br>• Thrombosis<br>• Air leakage<br>• Pneumonia<br>• Rebleeding<br>• Hemorrhage<br>• Infection<br>• Hoarseness<br>• Other	Frequency (%)	Frequency (%)	Frequency (%)	Frequency (%)
Type of hospital room last hospitalization<br>• 1-person patient room<br>• 2-person patient room<br>• 3-person patient room<br>• 4-person patient room<br>• More than 4 persons	Frequency (%)	Frequency (%)	Frequency (%)	Frequency (%)
Number of hospitalizations in life	Mean (SD)	Mean (SD)	Mean (SD)	Mean (SD)

Table 4 Baseline data – self reported psychophysical well-being

	**Intervention 1**	**Intervention 2**	**Intervention 3**	**Intervention 4**
General health (1 item)	Mean (SD)	Mean (SD)	Mean (SD)	Mean (SD)
General mental health (1 item)	Mean (SD)	Mean (SD)	Mean (SD)	Mean (SD)
Initial affective state (10 items)	Mean (SD)	Mean (SD)	Mean (SD)	Mean (SD)

Inc

Baseline characteristics will be presented using descriptive statistics, i.e. for quantitative parameters the mean and standard deviation (or SEM) or median and minimum/maximum with number of valid observations, depending on normality of data. Mean and median will be reported

to a precision of one decimal place more than the individual measurements; standard deviation will be reported to a precision of two decimal places more than the individual measurements; and min/max will have the same precision as the individual measurements. For qualitative parameters (categorical or ordered), frequency counts and percentages of each category will be calculated by intervention. Percentages will be reported up to 1 decimal place. Baseline characteristics will be summarized overall, and by treatment when applicable.

4.5 Outcomes

4.5.1 General considerations

There will be no missing values on any of the self-reported items, because the online questionnaire procedure requires an answer to all questions to complete the questionnaire. Missing values are only possible when participants stop halfway through the questionnaire.

4.5.2 Primary outcomes

Analysis of covariance (ANCOVA) will be conducted to test the main effect of the interventions on patients' anxiety and stress as well as the interaction effect of intervention and environmental sensitivity on patients' anxiety and stress. It is expected that participants who score higher on environmental sensitivity will react stronger to the intervention. Therefore, we will test whether environmental sensitivity moderates the effect of the intervention on each dependent measure (perceived anxiety, sense of control, positive distraction, social support, atmospherics of the patient room, and pleasantness of the room). Hayes Process Macro (Hayes, 2013) will be used to test the moderation effect.

4.5.3 Secondary outcomes

Separate linear regression analyses will be conducted to test the association of the intervention on sense of control, positive distraction, social support, atmospherics of the patient room, and pleasantness of the room.

It is expected that patients perceive less anxiety when they perceive more sense of control, positive distraction, social support, atmospherics of the patient room, and pleasantness of the room. Therefore, we will test for the indirect effect of the intervention on anxiety through the five mediators. This mediation analysis includes intervention as independent variable, and sense of control, positive distraction, social support, atmospherics of the patient room, and pleasantness of the room as mediator, and perceived anxiety as outcome. Hayes Process Macro (Hayes, 2013) will be used to test the mediation effect.

4.6 Statistical methods used for efficacy

For quantitative parameters, descriptive statistics will be mean, standard deviation (or SEM), or median and minimum-maximum, and number of valid observations by intervention group, depending on normality of data. Mean or median will be reported to a precision of one decimal place more than the individual measurements; standard deviation will be reported to a precision of two decimal places more than the individual measurements; and minimum-maximum will have the same precision as the individual measurements.

For qualitative parameters (categorical or ordered), frequency counts and percentages of each category will be calculated by intervention group. Percentages will be reported up to 1 decimal place. Where applicable, 95% confidence intervals around percentages will be calculated.

A 2-tailed P-value less than 0.05 indicates statistical significance. All analyses will be performed using SPSS.

5. Presentation of results

Statistical output will be generated in accordance to this Statistical Analysis Plan and results will be presented in accordance with international standards for reporting clinical studies, as a manuscript to be submitted to a medical journal (CONSORT)

In addition, results of all analyses will be presented in more detail using predefined tables of baseline data, efficacy results, as specified in the Tables and Figure below.

Table 5 Descriptive statistics

Dependent variables	Intervention 1	Intervention 2	Intervention 3	Intervention 4
Patients' anxiety				
Patients' stress				
Sense of control				
Positive distraction				
Social support				
Atmospherics of the patient room				
Perceived pleasantness				

Table 6 Results Mediation Analysis on patients' anxiety and sense of control

	Patients' anxiety			Sense of control		
	Coef.	SE	p	Coef.	SE	p
Intervention group						
Sense of control						

Table 7 Results mediation analysis on patients' anxiety and positive distraction

	Patients' anxiety			Positive distraction		
	Coef.	SE	p	Coef.	SE	p
Intervention group						
Positive distraction						

Table 8 Results mediation analysis on patients' anxiety and social support

	Patients' anxiety			Social support		
	Coef.	SE	p	Coef.	SE	p
Intervention group						
Social support						

Table 9 Results mediation analysis on patients' anxiety and perceived pleasantness

	Patients' anxiety			Perceived pleasantness		
	Coef.	SE	p	Coef.	SE	p
Intervention group						
Perceived pleasantness						

Figure 2 Presentation interaction effect of intervention and environmental sensitivity on patients' anxiety

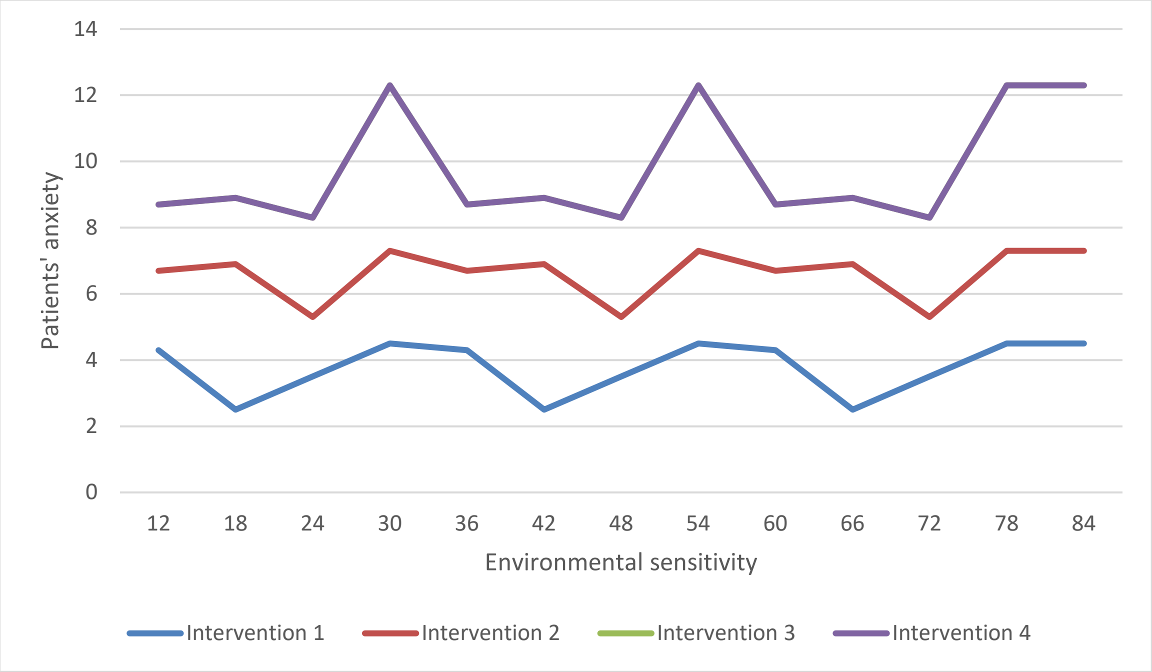

6. References

Andrade, C. C. *et al.* (2017) 'Do the hospital rooms make a difference for patients' stress? A multilevel analysis of the role of perceived control, positive distraction, and social support', *Journal of Environmental Psychology*, 53, pp. 63–71.

Andrade, C. C. and Devlin, A. S. (2015) 'Stress reduction in the hospital room: Applying Ulrich's theory of supportive design', *Journal of Environmental Psychology*, 41, pp. 125–134.

Aron, E. N. and Aron, A. (1997) 'Sensory-Processing Sensitivity and Its Relation to Introversion and Emotionality', *Journal of Personality and Social Psychology*, 73(2), pp. 345–368. doi: 10.1037/0022-3514.73.2.345.

Brysbaert, M. (2019) 'How many participants do we have to include in properly powered experiments? A tutorial of power analysis with some simple guidelines', *Journal of Cognition*, 2(1), pp. 1–38.

Hayes, A. F. (2013) *Introduction to mediation, moderation, and conditional process analysis*. New York: Guilford publications.

Marteau, T. M. and Bekker, H. (1992) 'The development of a six-item short-form of the state scale of the Spielberger state-trait anxiety inventory (STAI)', *Britisch Journal of Clinical*

Psychology, 31, pp. 301–306.

Pluess (2013) ‘Sensory-Processing Sensitivity: A potential mechanism of differential susceptibility’, in *Presented at the Society for Child Development*. Seattle, WA.

Suess, C. and Mody, M. (2018) ‘The influence of hospitable design and service on patient responses’, *Service Industries Journal*, 38(1–2), pp. 127–147. doi: 10.1080/02642069.2017.1385773.
